# Biotransformation of ketamine in terminal in vivo experiments under chronic intermittent hypoxia conditions and the role of AhR

**DOI:** 10.1007/s00204-025-04044-w

**Published:** 2025-04-19

**Authors:** António B. Pimpão, Luísa Teixeira-Santos, Nuno R. Coelho, Maria João Correia, Judit Morello, Alexandra M. M. Antunes, Emília C. Monteiro, Sofia A. Pereira

**Affiliations:** 1 iNOVA4Health, NOVA Medical School | Faculdade de Ciências Médicas, NMS|FCM, Universidade NOVA de Lisboa, Lisboa, Portugal; 2 Centro Clínico Académico de Lisboa (CCAL), Lisboa, Portugal; 3 Centro de Química Estrutural, Institute of Molecular Sciences, Departamento de Engenharia Química, Instituto Superior Técnico (IST), Universidade de Lisboa, Lisboa, Portugal

**Keywords:** Norketamine glucuronide, Hydroxynorketamine, Metabolism, Obstructive sleep apnea, Anesthesia, Aryl hydrocarbon receptor

## Abstract

We were pioneers in describing aryl hydrocarbon receptor (AhR) activation by chronic intermittent hypoxia (CIH) in a rat pre-clinical model. This model mimics hypertension (HTN) secondary to obstructive sleep apnea, enabling longitudinal investigation of hypertension development. Concerns about the influence of barbiturates on AhR-regulated enzymes led us to opt for ketamine/medetomidine anesthesia in terminal in vivo experiments. However, the biotransformation and the metabolomic pathways of ketamine in CIH conditions, which is associated to AhR overactivation, are yet to be disclosed. A rat model of CIH was used, with experimental groups defined based on the duration of CIH exposure. Ketamine/medetomidine (75/0.5 mg/kg) was administered intraperitoneally as terminal anesthetic. Metabolomic strategies were used to reveal the profiles of ketamine and its metabolites in liver and kidney tissues, uncovering six metabolites, including the first report of norketamine glucuronide formation in the liver. While PCA analysis revealed similar ketamine metabolite fingerprints in normoxia and CIH, a predominance of hydroxynorketamine over norketamine was observed in CIH condition. A consistent association between norketamine, hydroxyketamine and the metabolome was found in both normoxia and CIH conditions. The AhR antagonist CH-223191 (5 mg/kg) influenced hydroxynorketamine glucuronidation in the liver. No changes in medetomidine biotransformation were detected. Overall, these findings expand the knowledge of ketamine metabolism and its tissue-dependence. The results emphasize the importance of considering how ketamine biotransformation may differ between control and experimental conditions in metabolic studies, particularly in chronic intermittent hypoxia conditions. The role of AhR in ketamine biotransformation is herein described for the first time.

**Supplementary Information:**

The online version contains supplementary material available at 10.1007/s00204-025-04044-w.

## Introduction

We have been studying the link between the aryl hydrocarbon receptor (AhR) activation and the hypertension (HTN) secondary to obstructive sleep apnea (OSA). Most of our data come from pre-clinical studies wherein we use a rat model subjected to a chronic intermittent hypoxia (CIH) paradigm which mimics mild OSA (Diogo et al. 2015; Coelho et al. 2020; Arnaud et al. 2023). The CIH and its hypoxia/reoxygenation cycles activate several molecular pathways like NADPH oxidase and xanthine oxidase, triggering an increased ROS production. The oxidative stress thereby formed damages several organs and tissues, an effect potentiated also by inflammatory pathway activation (NF-kB) (Ryan et al. 2005). CIH effects are time- and tissue-dependent (Correia et al. 2021). Also, tissue hypoxia, as described for kidney, is present following exposure to long-term but not short-term intermittent hypoxia in the rat (O’Neill et al. 2019). With the chronicity of CIH, the animals develop arterial HTN, and we were the first to demonstrate that CIH activates the AhR signaling, particularly in the kidney, and a role for AhR signaling in CIH-induced arterial hypertension (Coelho et al. 2020; Correia et al. 2021; Pimpão et al. 2023).

The AhR was classically described as a ligand-activated transcription factor crucial in mediating the body’s response to environmental toxins, such as dioxins and polycyclic aromatic hydrocarbons (Coelho et al. 2021, 2022). When activated by these ligands, the AhR influences the expression of several genes, including those involved in xenobiotic metabolism (Coelho et al. 2022). Beyond this, the AhR integrates environmental, dietary, microbial, and metabolic cues to regulate a wide array of biologic functions (Coelho et al. 2022).

In this pre-clinical research about the activation of the AhR under CIH conditions, we used toxic doses of ketamine as anesthetic in terminal procedures. This choice was made because barbiturates, which are known regulators of xenobiotic biotransformation (Zaher et al. 1998; Madan et al. 2003)—a well-established function of AhR (Coelho et al. 2021, 2022)—could potentially interfere with our study outcomes.

Despite ketamine’s use as an anesthetic and analgesic, along with its potential for novel indications (Popova et al. 2019; Daly et al. 2019), the biotransformation and metabolomic alterations induced by ketamine, particularly under conditions of CIH, remain uncharacterized. Additionally, ketamine’s suitability for studies focusing on the AhR is yet to be explored.

Recognizing this knowledge gap and the critical need for controlling the impact of different experimental conditions on drugs biotransformation, we considered that our experimental setup presented an opportunity to generate data elucidating the pharmacologic pathways of ketamine and ketamine’s application in metabolomic in vivo studies. Specifically, this study aimed to assess the biotransformation and metabolomic alterations induced by ketamine/medetomidine combination, when used as anesthesia for terminal procedures of rodents exposed to varying durations of CIH, and also the impact of AhR.

## Materials and methods

### Animals

Male Wistar Hannover [Crl:WI (Han)] rats (*Rattus norvegicus* L.) were obtained from the animal facility of NOVA Medical School. The cages had corncob bedding and were changed weekly. The animals were maintained under standard laboratory conditions, i.e., artificial 12 h light/dark cycles (lights on at 8 a.m.), at room temperature (22 ± 2.0 °C) and a relative humidity of 60 ± 10%. Rats were given standard maintenance laboratory diet, in form of dried pellets (Special Diet Service-SDS diets RM1) and reverse osmosis water in drinking bottles, both ad libitum.

All applicable institutional and governmental regulations concerning the ethical use of animals in research were followed, according to the NIH Principles of Laboratory Animal Care (NIH Publication 85-23, revised 1985), the European guidelines for the protection of animals used for scientific purposes (European Union Directive 2010/63/EU) and the Portuguese regulation and laws on the protection of animals used for scientific purposes (Law nº 113/2013). All experimental procedures were approved by the Ethical Committee of the NOVA Medical School for the animal care and use in research (protocol nº 15/2017/CEFCM) and by the Portuguese General Directorate for Animal Health (DGAV—*Direcção-Geral de Alimentação e Veterinária*).

#### Chronic intermittent hypoxia (CIH) animal model

The paradigm of CIH employed here was previously described and validated by our group (Diogo et al. 2015; Coelho et al. 2020, 2021; Pimpão et al. 2023) and others (Diogo and Monteiro 2014; Arnaud et al. 2023). Briefly, the animals were housed in a eucapnic atmosphere within medium A-chambers (A-60274-P, Biospherix Ltd) and exposed to 10.5 h of intermittent hypoxia (IH) per day. The chambers were equipped with gas injectors and sensors to monitor O_2_ and CO_2_ levels, ensuring the accuracy of the CIH cycles. Oxygen concentration inside the chambers was regulated by electronically controlled solenoid switches, which adjusted the flow of 100% N_2_ and 100% O_2_ gas through a three-channel gas mixer, gradually reducing the oxygen from 21 to 5% (OxyCycler AT series, Biospherix Ltd.). A CIH cycle involved the infusion of 100% N_2_ for 3.5 min to rapidly reduce O_2_ concentration to ~ 5–6%, followed by an infusion of 100% O_2_ for 7 min to restore O_2_ to normal levels (~ 21%). Each cycle lasted 10.5 min (5.6 CIH cycles per hour) and occurred during the inactive period of the animals (9:30 AM–8:00 PM). During the remaining hours of the day, the chambers were ventilated with a continuous flow of room air (21% O_2_).

#### Study design

Male Wistar rats were exposed to CIH for 2, 3, 5 and 9 weeks (*n* = 5 per group) to evaluate the temporal impact of CIH in ketamine biotransformation. Animals maintained under normoxic conditions (Nx) (79% N_2_ and 21% O_2_) for each time point of the study were used as controls (*n* = 5 per group).

To evaluate the role of AhR signaling in ketamine biotransformation, animals were subjected to CIH for 3 weeks, starting on the 22^nd^ day, daily administration of CH-223191 (5 mg/kg/day in vegetable oil by oral gavage). This design was selected because in the first 3 weeks, animals present an increase in blood pressure with the duration of CIH exposure, reaching a plateau up to week 5. Rats were maintained under this treatment and in CIH condition for the subsequent 2 weeks. Animals under Nx or Nx treated with CH-223191 were used as controls (*n* = 3 per group).

#### Ketamine/medetomidine administration and tissue collection

At the end of the experiments, rats were intraperitoneally injected with a solution of ketamine (75 mg/kg body weight; Imalgene 1000, Boehringer Ingelheim Animal Health, France) and medetomidine (0.5 mg/kg body weight; Domitor, Pfizer Animal Health, New Zealand). After the anesthetics administration, blood was drawn by cardiac puncture without thoracotomy and tissues were collected. Death was confirmed by cervical dislocation. Both liver and kidney cortex tissues were then collected, snap-frozen in liquid nitrogen and all samples were stored at –80 °C until analyzed.

### Metabolomics analysis

#### Metabolite extraction

Before metabolite extraction, samples were randomized. Briefly, 200 µL of cold methanol/water (75%) solution was added to each 40 mg of tissue. Samples were vortexed and sonicated using an ultrasound bath at 4 °C for 60 min to enhance metabolite extraction. Then, samples were centrifuged at 13,200 rpm for 10 min at 4 °C and 160 µL of the supernatant was transferred to a new microcentrifuge tube for dry vacuum. Dried samples were reconstituted with 10% acetonitrile in water in a final volume of 600 µL. A volume of 5 µL of each reconstituted sample was pooled together (quality control pool; QCpool).

#### Liquid chromatography–high-resolution mass spectrometry (LC-HRMS)

Samples were analyzed by liquid chromatography (Elute UHPLC, Bruker, Bremen, Germany) interfaced with a Bruker Impact II quadrupole time-of-flight mass spectrometer equipped with an electrospray source (Bruker Daltoniks, Bremen). Chromatographic separation was performed on a LUNA C18 column (150 mm × 2.0 mm; 3.0 μm, Phenomenex) (Phenomenex) at 45 °C. A flow rate of 150 μL/min was used and the eluent system consisted on formic acid 0.1% (phase A) and acetonitrile (phase B), with the following gradient program: 1.5 min 5% phase B, then in 13 min to 100% phase B, held for 3.5 min at 100% phase B, subsequently in 0.25 min to 5% phase B and held for 1.75 min at 5% phase B. Calibration was performed by high-precision calibration mode (HPC) on the internal standard segment, consisting of sodium formate solution introduced at the beginning of each analysis. The mass spectrometer was operated in positive ionization mode on the full scan mode and data were acquired in the mass range from *m*/*z* 50 to 1000 with a spectra rate of 1 Hz. The capillary was set at 4.5 kV, the End Plate offset at 500 V, the Nebulizer gas (N_2_) at 40 psi and the Dry gas (N_2_) at 8 L/min at 200 °C. To evaluate the performance of the instrument, QCpools were injected with every 7 samples. QCpools were also acquired in DDA mode on the same instrument, with an isolation window of 0.5, acquisition rate of 3 Hz and a fixed cycle time of 3 s.

#### Targeted peak detection of ketamine and ketamine metabolites and medetomidine

Ketamine, ketamine metabolites and medetomidine were identified from the expected *m/z* values of the precursor ions and the product ions published elsewhere (Turfus et al. 2009; Bijlsma et al. 2011; Dinis-Oliveira 2017) and/or available in Human Metabolome Database. LC–MS files were converted to mzXML files using the ProteoWizard MSConvert software (Chambers et al. 2012). A targeted analysis was then performed with the open-source software MZmine3 (Katajamaa et al. 2006; Pluskal et al. 2010), which consisted of target peak detection and peak matching.

Targeted peak detection was performed with the list of the corresponding *m*/*z* and retention time values for each precursor compound (Table S1) and the following parameters: shape tolerance = 50%, noise level = 100, *m*/*z* tolerance = 0.005 Da or 15 ppm and retention time tolerance = 0.5 min. Peak matching among samples was performed using the Join aligner algorithm with *m*/*z* tolerance = 0.005 Da or 15 ppm, retention time tolerance = 0.5 min, weight for *m*/*z* and retention time = 1 and required same identification.

#### Untargeted LC–MS data preprocessing

Kidney and liver LC–MS data were preprocessed separately using XCMS 3.6.0 using XCMS 3.6.0 (Smith et al. 2006; Tautenhahn et al. 2008) in R environment (http://www.r-project.org/, R version 4.2.2). Preprocessing consisted of peak picking, retention time alignment, peak grouping and gap filling. Peak picking was performed with the centwave algorithm (Tautenhahn et al. 2008) and the following parameters: ppm = 20, peak width = 10–50 s, prefilter = 6, 10,000. Retention time alignment was performed against the average of the QCpools using the Obiwarp method with a *m*/*z* width = 0.01. Peak grouping was performed with the following peak density parameters: band width = 30, *m*/*z* width = 0.01, minimum fraction = 0.5. Gap filling was performed with a fixed retention time deviation = 15 s. Ions were further filtered according to the relative standard deviation of ions in QCpools (≤ 30%). The final dataset contained a total of 399 and 494 features (peaks with specific retention time and *m*/*z* values) with their corresponding *m*/*z*, retention time and peak area for the kidney and liver, respectively. The signals corresponding to ketamine, medetomidine and related compounds (isotopes, metabolites, fragments, adducts) were excluded from the dataset. To do that, the features corresponding to ketamine and medetomidine were identified and correlated with the rest of the ions present in the dataset. Those highly correlated features (correlation coefficient ≥ 0.8) were excluded from the dataset. Data were normalized by total area and centered and unit-variance scaled before statistical analysis.

### Statistical analysis

Results are presented as mean ± standard error of the mean (S.E.M.). The normality of distributions was assessed using the Shapiro–Wilk test and comparison of unpaired data was performed using Student’s *t* test or Mann–Whitney test, using GraphPad Prism software version 8 (GraphPad Software, San Diego, CA, USA). *p* values < 0.05 were considered significant. Additionally, Principal Component Analysis (PCA), Partial Least Square Analysis (PLS) and Partial Least Square Discriminant Analysis (PLS-DA) were performed using SIMCA software (MKS Umetrics, Umeå, Sweden). Details on the datasets used to perform each analysis are provided in figure’s captions.

## Results

Ketamine (Fig. S1) and medetomidine (Fig. S2) were found in both liver (Figs. S3a and S4A–D) and kidney tissues (Figs. S3 and S4E–H). The comparison of disposition of both drugs between the two conditions, normoxia (Nx) and chronic intermittent hypoxia (CIH) was conducted across various study timepoints and the animal-to-animal variability was calculated for the Nx (*n* = 20) and CIH (*n* = 20) groups. When comparing all normoxia (Nx) groups and all CIH groups, we observed more consistent results, with lower animal-to-animal variability in the kidney than in the liver for both ketamine and medetomidine. Overall, the biggest variability between animals was found for ketamine exposure in the liver (95%) and the lowest for ketamine in the kidney (31%) (Fig. S5). While no changing pattern was observed with the chronicity of intermittent hypoxia, the disposition of ketamine was lower in 2 weeks of CIH, in the liver, than in Nx controls (Fig. S3A). As for the medetomidine, in animals exposed to CIH for 3 weeks, a higher disposition of the drug was observed in the kidney compared to controls (Fig. S4F).

**Fig. 1:**
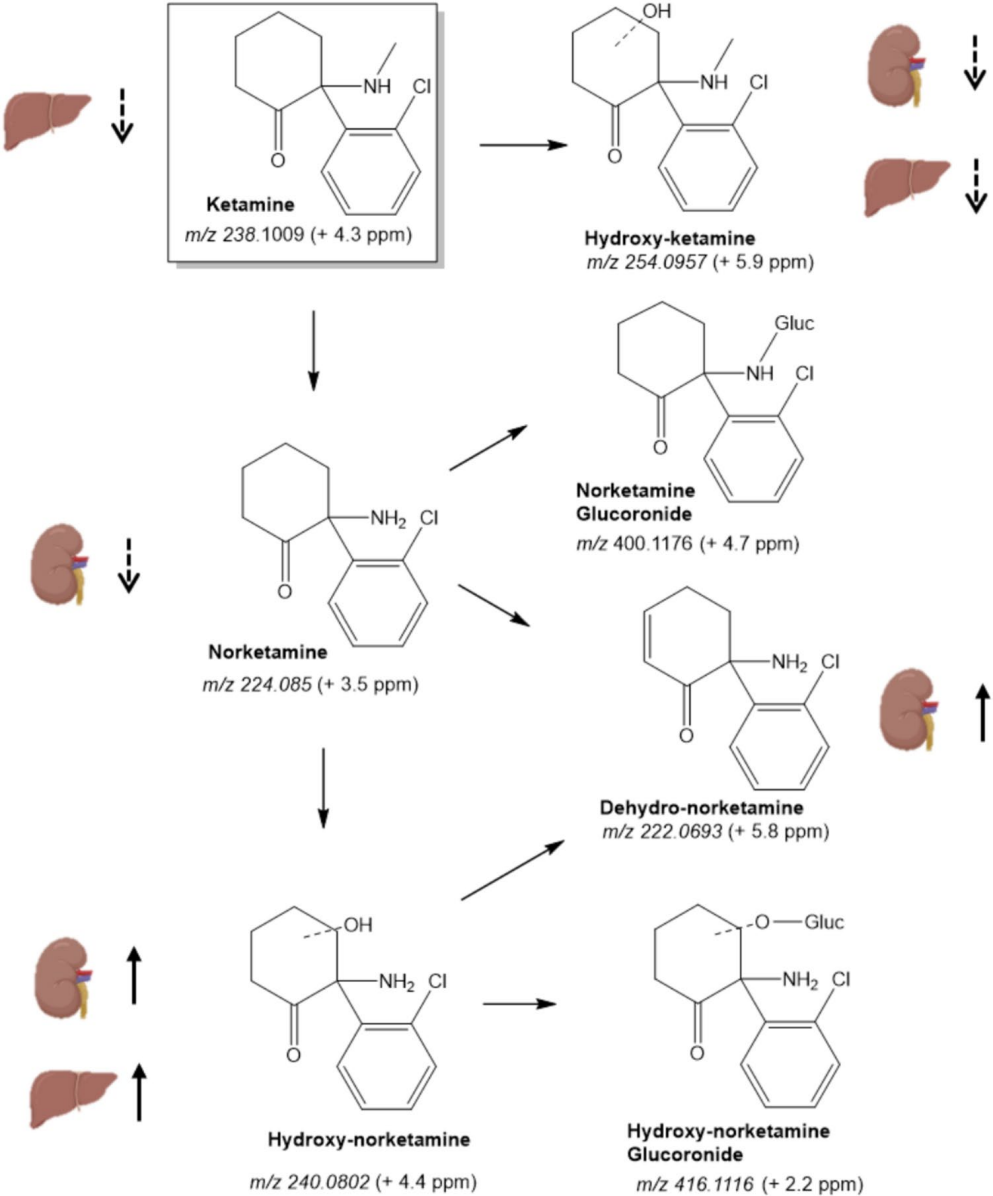
Ketamine biotransformation pathways and metabolites found in liver and kidney tissues. Arrows next to each organ indicate the direction of change for each metabolite in chronic intermittent hypoxia condition. Dashed arrows show the effect of short-term intermittent hypoxia (2–3 weeks), and the full arrows show the effect of long-term intermittent hypoxia (5–9 weeks). **↑**: increase; **↓**: decrease

**Fig. 2:**
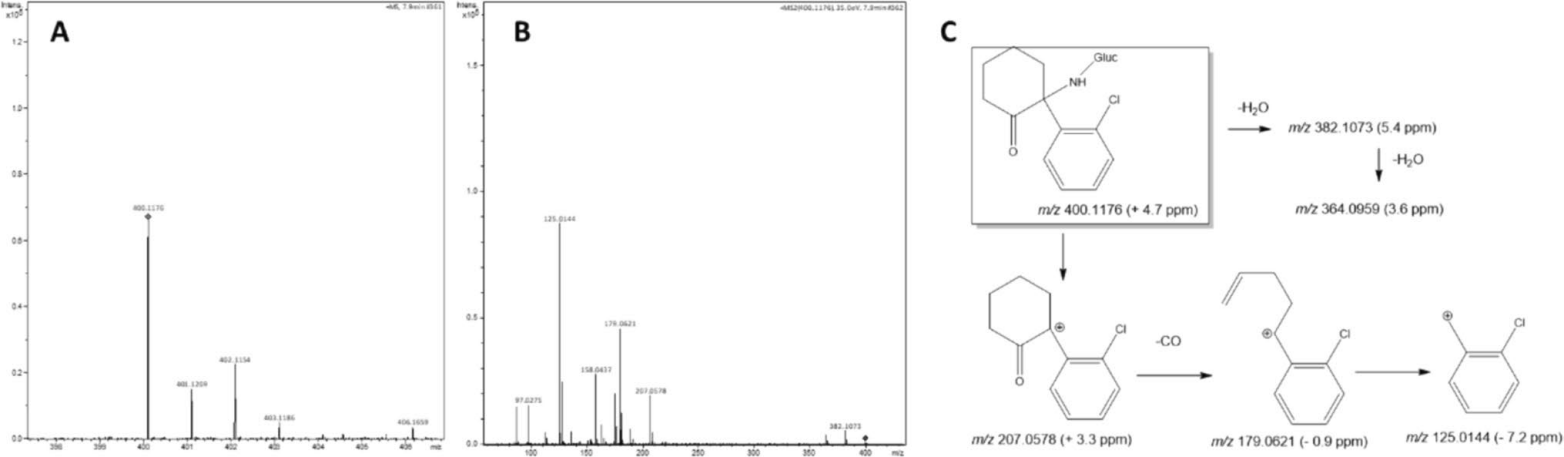
HRMS-ESI (+) obtained for norketamine glucuronide. **A** Full-scan mass spectrum; **B** Tandem mass spectrum and **C** Potential structures of the main fragment ions

No metabolites of medetomidine were detected in either tissue. As for ketamine, a total of six ketamine metabolites were identified (schematized in Fig. 1). These include the Phase I metabolites norketamine, hydroxyketamine, hydroxynorketamine, dehydronorketamine (Figs. S6–S9), but also the Phase II metabolites hydroxynorketamine glucuronide (Fig. S10) and norketamine glucuronide. Multiple signals consistent with isomeric structures of hydroxyketamine and hydroxynorketamine metabolites, stemming from hydroxylation at aromatic and allylic positions, as recently reported (Vallianatou et al. 2024), were also detected in our study. However, due to the absence of synthetic standards and the inability to distinguish these structures based solely on their tandem mass spectra, we decided to use the overall area of signals with the same *m*/*z* for our analysis. Noteworthy, to the best of our knowledge, the extensive MS/MS fragmentation analysis allowed for the conclusive identification of norketamine glucuronide, presented in Fig. 2, for the first time, albeit a glucuronide metabolite with the same *m*/*z* was recently reported in the mammalian brain (Vallianatou et al. 2024).

The metabolites of ketamine were evaluated across the different durations of CIH (2, 3, 5 and 9 weeks) and compared to Nx controls, as depicted in Figs. S11–S14 for Phase I metabolites, and in Figs. S15 and S16 for Phase II metabolites. In Fig. 1 we added an overall view of the variations in metabolites.

**Fig. 3:**
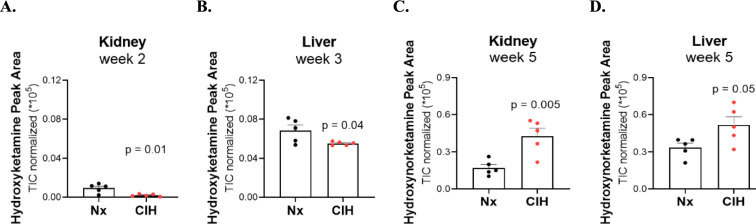
Hydroxyketamine and hydroxynorketamine disposition in liver and kidney tissues. Hydroxyketamine disposition with short-term (week 2 (**A**) and week 3 (**B**)), and hydroxynorketamine disposition with long-term (week 5 (**C**) and (**D**)) exposure to chronic intermittent hypoxia (CIH) vs normoxia (Nx) paradigms. The represented *p* values are derived from unpaired *t* tests. *n* = 5 animals per group. *AU* arbitrary units, *TIC* total ion chromatogram

For Phase I metabolites, the decrease found in hydroxyketamine in both tissues in shorter exposure to CIH (Fig. 3A, B), with the subsequent increase in hydroxynorketamine in longer CIH exposure (Fig. 3C, D), could be indicative of a shift of ketamine biotransformation to norketamine pathway under CIH condition. This observation was consistent, as it was observed in both tissues. No changes were observed for the glucuronides identified (Figs. S15 and S16).

AhR is a ligand-activated transcription factor that presents constitutive activation in normoxia conditions. AhR activation is assessed by measuring the expression of its hallmark target gene, CYP1 A1, which is why the term “AhR-CYP1 A1 activation” is commonly used. Employing the described CIH paradigm, we previously reported that AhR-CYP1 A1 remained under-activated up to the third week of CIH exposure but became overactivated thereafter (Correia et al. 2021).

Since AhR is a ligand-activated transcription factor involved in the regulation of xenobiotic metabolism, and blocking AhR can influence the expression of various genes involved in drug biotransformation, we also investigated ketamine biotransformation in the presence of the most widely used AhR antagonist, the CH-223191 (Coelho et al. 2022). We profiled metabolites of ketamine in the Nx group with or without CH-22391 and compared the impact of CH-223191 in CIH vs. Nx. PCA was performed to evaluate the impact of CIH and AhR blockage on Ketamine and ketamine metabolites. Overall, no major differences were found in the pattern of ketamine metabolites in CIH condition (Figs. S17 and S18), nor in the presence of the AhR antagonist (Fig. S19). PLS-DA confirmed the results observed in PCA (data not shown).

**Fig. 4:**
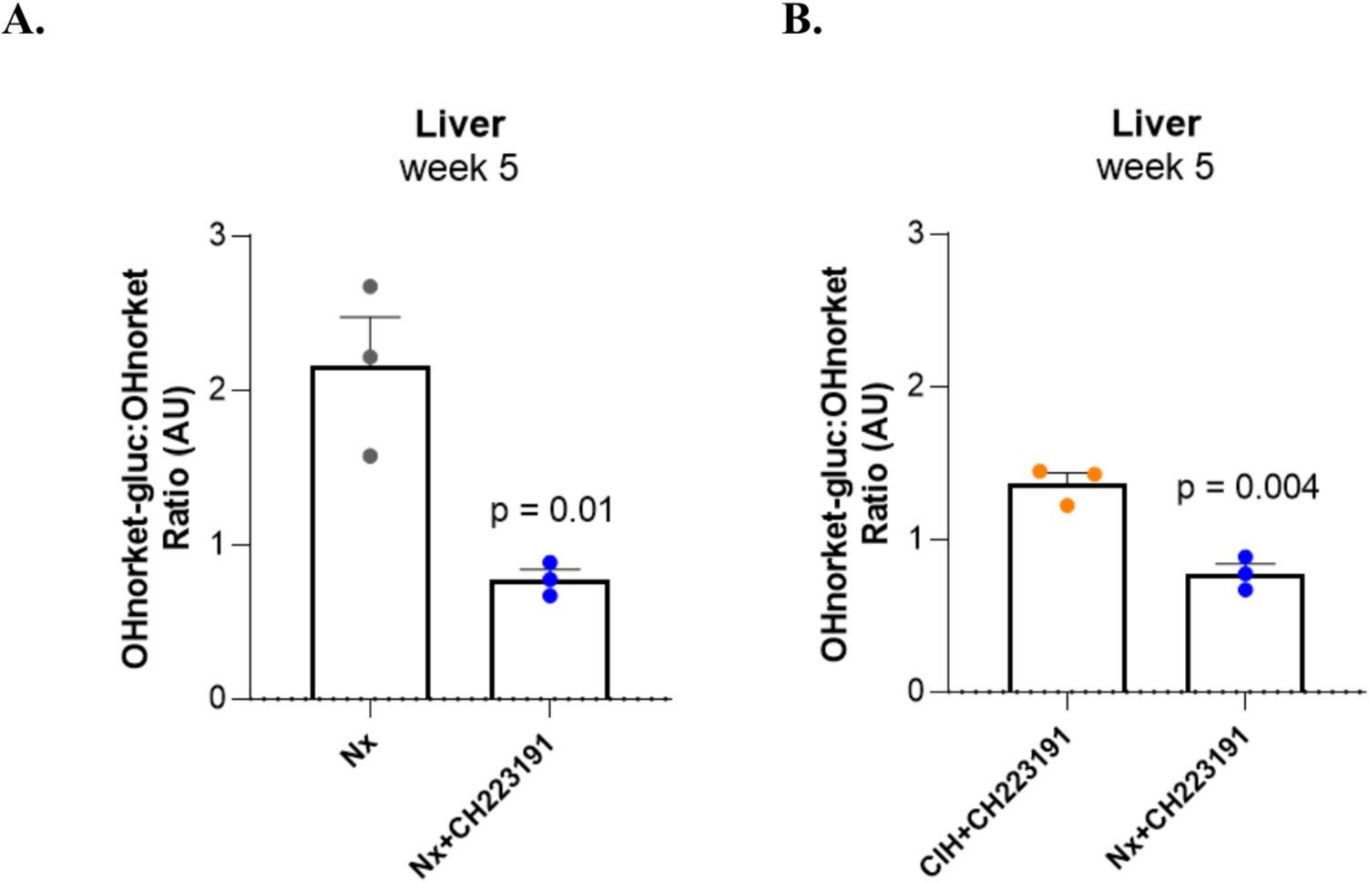
Effect of the AhR antagonist CH-223191 in hydroxynorketamine glucuronidation in the liver: metabolite ratios **(A)** in Nx and **(B)** in CIH (OHnorket-gluc:OHnorket). The represented *p* values are derived from unpaired t tests. *n* = 3 animals per group. *AU* arbitrary units

As less information is available regarding the regulation of Phase II enzymes by AhR, we further investigated the glucuronide metabolites of ketamine in animals treated with the AhR antagonist (Fig. S20). There was a decrease in hydroxynorketamine glucuronidation in the liver under Nx in the presence of the antagonist (Fig. 4A), comparatively to non-treated animals. This decrease of hepatic hydroxynorketamine glucuronidation, prompted by the AhR antagonist in Nx, was also observed when compared with the use of AhR antagonist in CIH (Fig. 4B). This elicits a differential effect of AhR modulation in ketamine glucurono-conjugation under Nx and CIH conditions. No influence of the CH-223191 in glucurono-conjugation of norketamine was observed in the liver (Fig. S20A, B). Additionally, neither hydroxynorketamine glucuronide nor norketamine glucuronide was detected in the kidney of CH-223191-treated animals.

We also investigated whether there would be an association between the anesthetics and the endogenous metabolome through PLS analysis, treating medetomidine, ketamine or each of the metabolites of ketamine as the dependent variable (Table S2). The most relevant metabolic features for each model were ascertained as those having a variable importance on the projection (VIP) value > 1.50 and a correlation coefficient *p*(corr) value >| 0.75|. The number of metabolic features (FT) is indicated in the table for the PLS models with *p* < 0.05; *n* = 20 per group. All the data for each model, including the number of components, R2X, R2Y, and Q2, are provided in Table S3.

The analysis yielded two primary observations. First, the number of identified metabolomic features was higher in Nx than in CIH condition, with the exception of hydroxynorketamine, which exhibited a slightly higher count in CIH. Second, the associations between both norketamine and hydroxyketamine with the metabolome remain consistent across all conditions (Table S2), suggesting a robust association that is independent of the tissue and unaffected by variation in oxygen.

## Discussion

This study advances our understanding of ketamine biotransformation, with its elucidation in CIH conditions. Six metabolites of ketamine were unveiled, both Phase I and Phase II, including the first description of norketamine glucuronide in the liver. The results reveal the impact of CIH and AhR signaling on ketamine biotransformation and provide insights into associations within tissue-specific metabolome.

Our study focused on two key organs involved in ketamine disposition: liver and kidney. The liver plays a central role in biotransformation (Edwards and Mather 2001), and the kidney clears ketamine from the bloodstream (Zanos et al. 2018), primarily eliminating it in glucuronide-conjugated forms (Dinis-Oliveira 2017). Ketamine use was reported to negatively affect renal function (Ou et al. 2020), dependent on dosage and specific experimental conditions (Curtis et al. 2011; Demirkiran et al. 2019; Ou et al. 2020). Ketamine’s biotransformation primarily involves hepatic N-demethylation, mediated by CYP enzymes (White et al. 1982; Hijazi and Boulieu 2002), with consequent formation of norketamine, its main metabolite. Other pathways of ketamine biotransformation are reviewed elsewhere (Mion and Villevieille 2013; Dinis-Oliveira 2017). In rats, high-dose ketamine administration (e.g., 80 mg/Kg/day for 4 days) increased hepatic CYP1A and CYP1B (known target genes of AhR signaling) (Chan et al. 2005) and CYP2B (Chan et al. 2005; Yeager et al. 2009) mRNA and protein levels, but the literature is not consensual (Loch et al. 1995; Chan et al. 2005). Moreover, an induction of Phase II hepatic drug-metabolizing enzymes, such as glutathione S-transferase (GST) and UDP-glucuronosyltransferase (UGT), has also been reported (Chan et al. 2008; Chang et al. 2009) However, a comprehensive understanding of ketamine biotransformation in different contexts and organ specificities is still lacking, particularly given the significance of ketamine in clinical practice and laboratory animal experimentation.

We have pioneered the description of biotransformation changes in the context of CIH (Diogo et al. 2015) and established a connection between CIH and AhR pathway activation, which led us to question whether CIH exposure can affect the anesthetic’s biotransformation. The use of a model of CIH, which is a clinical trait of OSA and recognized as the primary factor responsible for most of its comorbidities (Arnaud et al. 2023), has unique features for investigating context- and tissue-dependent biotransformation. CIH is associated with oxygen variations that burst oxidative stress and activate nuclear factor kappa-Β (NF-kβ) inflammatory pathways. Also, AhR shares with hypoxia-inducible factor (HIF) the dimerization partner (Arnaud et al. 2023). All of these factors are well-known to impact biotransformation. Given the high prevalence of OSA and its cardiovascular comorbidities (Yeghiazarians et al. 2021), the** s**tudy of drug biotransformation dependent on the CIH context is extremely significant.

Furthermore, the use of anesthetics in terminal in vivo studies is pivotal due to ethical considerations and for minimizing animal discomfort. However, the influence of these agents on biotransformation, especially in in vivo studies about specific metabolic pathways and their impact on metabolome, deserves careful attention, supported by evidence that justifies this selection. While barbiturates are known to influence it (Zaher et al. 1998), consensual evidence linking ketamine metabolism and AhR is currently lacking. Recognizing this gap in the current knowledge, our study investigated the putative impact of the anesthetic in terminal surgeries as a carefully controlled variable on tissue metabolomic outcomes, and their tissue-specific particularities in the particular case of CIH.

Herein, alterations in Phase I metabolites in CIH conditions are described for the first time, specifically a decrease in the pathways involved in ketamine hydroxylation and an increase in the norketamine hydroxylation. These changes might be suggestive of a predominance of norketamine pathway of the ketamine biotransformation under CIH conditions. Despite these changes, the overall metabolite fingerprint of ketamine in CIH remained consistent with Nx condition, suggesting a lack of significant impact on ketamine biotransformation pathways, both in short- and long-term CIH. This result supports ketamine use as a suitable option for metabolic studies in this model. Many in vivo studies with different research questions related to AhR employed a high dose of ketamine (50 or 100 mg/kg), often co-administered with xylazine (10 mg/kg), as anesthesia for the animal’s sacrifice (Baker et al. 2013a, b; Hsu et al. 2018). However, a number of other studies did not adopt this choice (Attignon et al. 2017; Huang et al. 2023; Shen et al. 2023).

The identification of ketamine and medetomidine in both hepatic and renal tissues revealed variability in ketamine disposition, especially within the liver. Acknowledging this variability makes it evident that the sample size should have been larger. Nevertheless, the recognition of this variability is demonstrated for the first time, providing a relevant insight for sample size calculations in future studies. Additionally, in the experiment to evaluate AhR and ketamine metabolism interplay, the sample dimension was limited by the prohibitive cost of CH-223191 (Dean et al. 2018; Coelho et al. 2020). However, this experience represented an exploratory opportunity, resulting in previously unknown discoveries. Using a pharmacologic approach, we demonstrate the control of AhR in the glucuronidation of hydroxynorketamine in the liver. Overall, this finding underscores a regulatory role of AhR signaling in Phase II biotransformation, which is less known and represents a novelty of this work.

A dosage of 75 mg/kg of ketamine was employed in this study, which we acknowledge as a limitation for the extrapolation of our findings to clinical practice. In humans, ketamine’s dose usually ranges from 1 to 4.5 mg/kg when used intravenously for the induction of anesthesia. Sub-anesthetic doses are indicated for analgesia or novel therapeutic indications that have been emerging, such as treatment-resistant depression (Andrade 2017; Schwenk et al. 2018). Therefore, although the dosage employed is not related to clinical practices, this study lays a foundation for future investigations into the clinical implications of altered drug metabolism, particularly in OSA patients. Moreover, these are indeed the dosages used in pharmacologic in vivo studies and hence this study provides valuable insights into the putative implications of acute ketamine use in experimental outcomes in the context of CIH.

The significance of differing impacts between the experimental and control conditions on anesthetic biotransformation and the metabolome is often underestimated in experimental design, yet these differences can putatively influence study outcomes. For instance, the importance of drug secondary metabolites as perpetrators in iatrogenic reactions and drug-drug interactions (VandenBrink and Isoherranen 2010; Baillie 2023) has been increasingly recognized in clinical practice. There are some clinically relevant examples in which Phase II metabolites are responsible for drug toxicity or interactions (Shitara et al. 2004; Scialis and Manautou 2016; Kahma et al. 2024). For example, glucuronidation of clopidogrel leads to inhibition of the biotransformation of repaglinide, as clopidogrel acyl-β-D-glucuronide is an inhibitor of CYP2 C8, the enzyme responsible for metabolizing repaglinide (Tornio et al. 2014). On the other hand, specific experimental conditions such as CIH can induce pharmacokinetic changes, as our groups has previously reported for carvedilol (Diogo et al. 2015).

Overall, this exploratory analysis of ketamine, its metabolites, and its association with metabolome indicates that there is still much to be discovered in the pharmacologic pathways of ketamine, and its tissue and context specificities. The work is relevant to decision-making in in vivo experimentation as the impact of experimental conditions on drugs pharmacokinetics must be carefully considered. Also, it is particularly timely as ketamine is returning to the spotlight with novel indications beyond anesthesia.

## Supplementary Information

Below is the link to the electronic supplementary material.

Supplementary file1 (DOCX 1993 KB)

## Data Availability

The datasets generated during and/or analysed during the current study are available from the corresponding author on reasonable request.
