# Supplementary material for: Biotransformation of ketamine in terminal in vivo experiments under chronic intermittent hypoxia conditions and the role of AhR
Source: Arch Toxicol. 2025 Apr 19;99(7):2835–44. doi: 10.1007/s00204-025-04044-w (PMC12198304; doi:10.1007/s00204-025-04044-w)
Supplement: Supplementary file 1 — Supplementary file1 (DOCX 1993 KB) [file 204_2025_4044_MOESM1_ESM.docx]

Biotransformation of ketamine in terminal *in vivo* experiments under chronic intermittent hypoxia conditions and the role of AhR

António B. Pimpão^1#^, Luísa Teixeira-Santos^1,2#^, Nuno R. Coelho^1^, Maria João Correia^1^, Judit Morelo^1^, Alexandra M. M. Antunes^3^, Emília C. Monteiro^1,2^, Sofia A. Pereira^1,2*^

^1^iNOVA4Health, NOVA Medical School | Faculdade de Ciências Médicas, NMS|FCM, Universidade NOVA de Lisboa, Lisboa, Portugal; ^2^Centro Clínico Académico de Lisboa (CCAL), Lisboa, Portugal; ^3^Centro de Química Estrutural, Institute of Molecular Sciences, Departamento de Engenharia Química, Instituto Superior Técnico (IST), Universidade de Lisboa, Lisboa, Portugal

*Correspondence: [sofia.pereira@nms.unl.pt](mailto:sofia.pereira@nms.unl.pt)

# Equal contribution

| **Table S1.** **List of medetomidine and ketamine metabolites analyzed in the targeted peak detection.** Structure, chemical formula, theoretical and observed mass-to-charge ratio (*m/z*) and retention time (Rt) values for each precursor compound are presented. | | | | | | |
| --- | --- | --- | --- | --- | --- | --- |
| **Chemical Structure** | **Name** | **Chemical Formula** | ***m/z* (theoretical)** [M+H]^+^ | ***m/z* (observed)** [M+H]^+^ | ***m/z* error** (ppm) | **Rt** (min) |
| 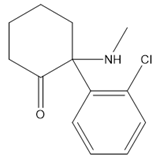 | Ketamine | C_13_H_16_ClNO | 238.0999 | 238.0985 | 5.87 | 7.56 |
| 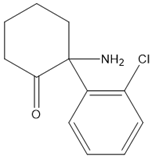 | Norketamine | C_12_H_14_ClNO | 224.0842 | 224.0828 | 6.25 | 7.44 |
| 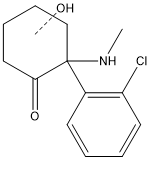 | Hydroxyketamine | C_13_H_16_ClNO_2_ | 254.0948 | 254.0973 | 9.84 | 6.67 |
| 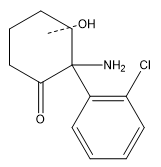 | Hydroxynorketamine | C_12_H_14_ClNO_2_ | 240.0791 | 240.0787 | 1.67 | 3.21 |
| 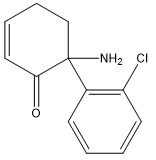 | Dehydronorketamine | C_12_H_12_ClNO | 222.0686 | 222.0673 | 5.85 | 7.11 |
| 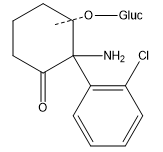 | Hydroxynorketamine Glucuronide | C_18_H_22_ClNO_8_ | 416.1107 | 416.1092 | 3.60 | 9.98 |
| 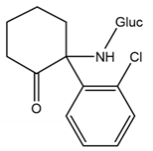 | Norketamine Glucuronide | C_18_H_22_ClNO_7_ | 400.1163 | 400.1136 | 6.75 | 7.87 |
| 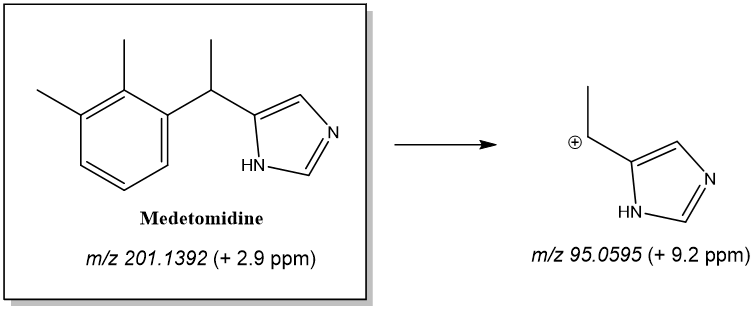 | Medetomidine | C_13_H_16_N_2_ | 201.1364 | 201.1374 | 4.97 | 8.6 |

| **Table S2. List of Partial Least Square Analysis (PLS) models,** built to investigate the association between medetomidine, ketamine, or each ketamine metabolite with the endogenous metabolome, in the liver and kidney, under normoxic (Nx) and chronic intermittent hypoxia (CIH) conditions. | | | | |
| --- | --- | --- | --- | --- |
| **Metabolite** | **Liver** | | **Kidney** | |
|  | **Nx** | **CIH** | **Nx** | **CIH** |
| **Medetomidine** | ns | ns | ns | ns |
| **Ketamine** | p<0.01  24 FT | p=0.02  17 FT | ns | ns |
| **Norketamine** | p<0.01  23 FT | p=0.03  9 FT | p<0.01  16 FT | p=0.01  11 FT |
| **Hydroxyketamine** | p=0.01  16 FT | p=0.03  10 FT | p<0.01  16 FT | p=0.03  13 FT |
| **Hydroxynorketamine** | ns | ns | p<0.01  10 FT | p<0.01  13 FT |
| **Dehydronorketamine** | ns | ns | p<0.01  32 FT | p=0.04  11 FT |
| **Hydroxynorketamine-glucuronide** | ns | ns | p=0.02  8 FT | ns |
| **Norketamine-glucuronide** | ns | (p=0.05) | (p=0.05) | ns |

The number of metabolic features (FT) having a Variable Importance on the Projection (VIP) value > 1.50 and a correlation coefficient p(corr) value > |0.75| (first component) are indicated in the table for the PLS models with p<0.05; n=20 per group. All the data for each model, including the number of components, R2X, R2Y, and Q2, are provided in **Table S2**.

**Table S3.** **Data on the Partial Least Square Analysis (PLS) models listed in Table S2.**

|  | **Liver** | | **Kidney** | |
| --- | --- | --- | --- | --- |
|  | **Nx** | **CIH** | **Nx** | **CIH** |
| Medetomidine | - | - |  |  |
| Ketamine | 2 components, R2X = 48%, R2Y = 83%, Q2 = 64% | 2 components, R2X = 46%, R2Y = 90%, Q2 = 59 | - | - |
| Norketamine | 3 components, R2X = 53%, R2Y = 98%, Q2 = 79% | 5 components, R2X = 68%, R2Y = 99%, Q2 = 83% | 2 components; R2X = 41%, R2Y = 91%, Q2 = 62% | 2 components, R2X = 39%, R2Y = 86%, Q2 = 61% |
| Hydroxyketamine | 3 components, R2X = 53%, R2Y = 98%, Q2 = 68% | 2 components, R2X = 46%, R2Y = 80%, Q2 = 55% | 2 components, R2X = 45%, R2Y = 93%, Q2 = 71% | 2 components, R2X = 31%, R2Y = 84%, Q2 = 45% |
| Hydroxynorketamine | - | - | 2 components, R2X = 46%, R2Y = 89%, Q2 = 74% | 2 components, R2X = 38%, R2Y = 92%, Q2 = 73% |
| Dehydronorketamine | - | - | 2 components, R2X = 46%, R2Y = 89%, Q2 = 67% | 2 components, R2X = 39%, R2Y = 80%, Q2 = 48% |
| Hydroxynorketamine-glucuronide | - | - | 2 components, R2X = 46%, R2Y = 84%, Q2 = 52% | - |
| Norketamine-glucuronide | - | - | - | - |

Nx, Normoxia condition; CIH, chronic intermittent hypoxia condition


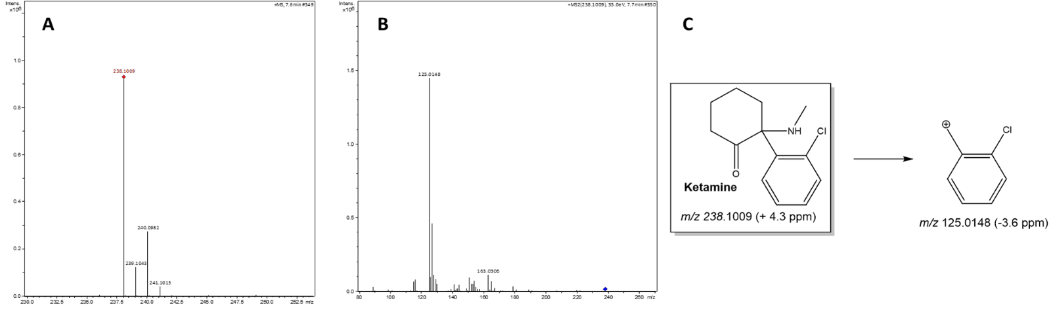


**Fig. S1.** **HRMS-ESI (+) obtained for ketamine.** **(A)** Full scan mass spectrum; **(B)** tandem mass spectrum and **(C)** potential structures of the main fragment ions


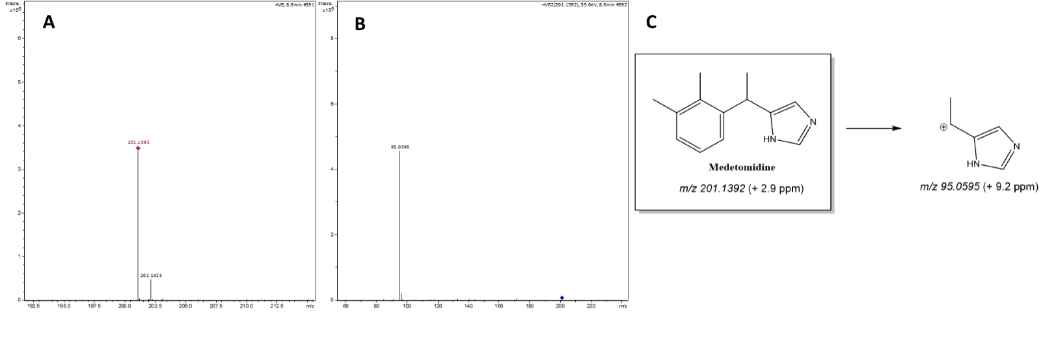


**Fig. S2.** **HRMS-ESI (+) obtained for medetomidine.** **(A)** Full scan mass spectrum; **(B)** tandem mass spectrum and **(C)** potential structures of the main fragment ions

| **A.**  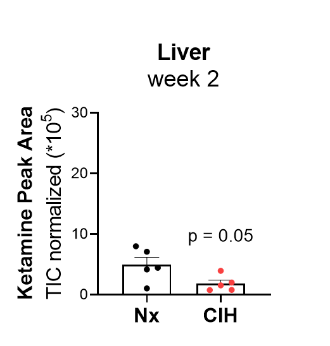 | **B.**  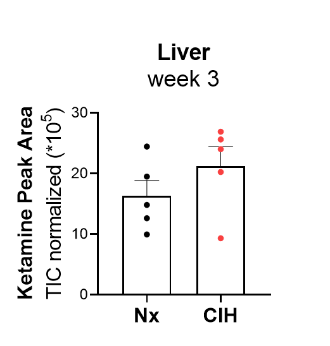 | **C.**  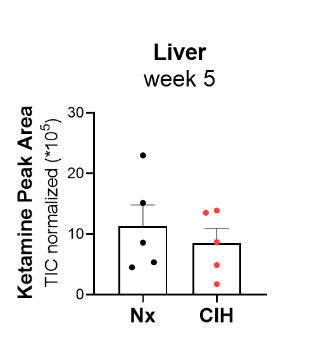 | **D.**  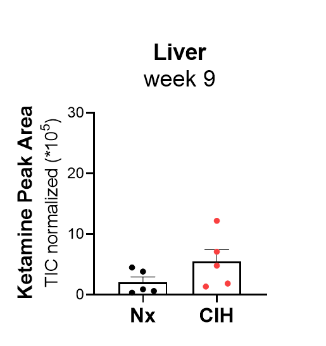 |
| --- | --- | --- | --- |
| **E.**  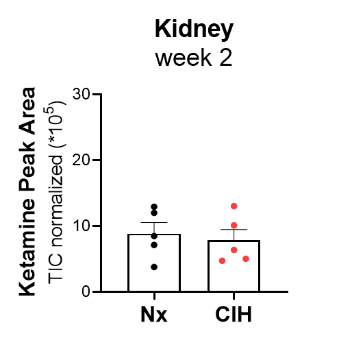 | **F.**  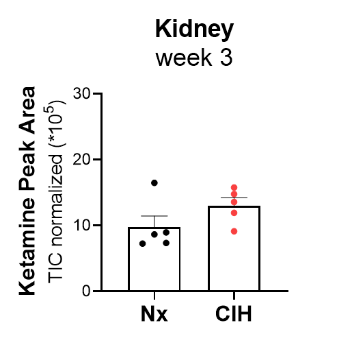 | **G.**  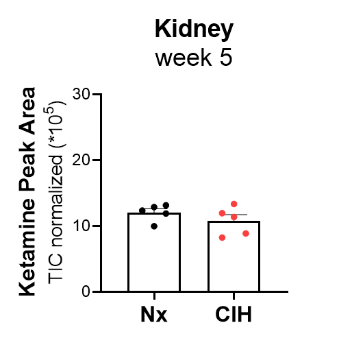 | **H.**  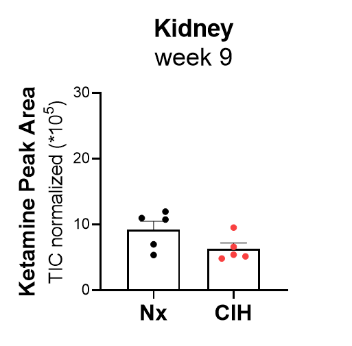 |

**Fig. S3. Ketamine disposition in liver and kidney tissues**, at **(A)** and **(E)** week 2, **(B)** and **(F)** week 3, **(C)** and **(G)** week 5, and **(D)** and **(H)** week 9 of exposure to chronic intermittent hypoxia (CIH) vs normoxia (Nx) paradigms. The represented *p*-value is derived from unpaired t-test. n=5 animals per group. **AU**, arbitrary units; **TIC**, total ion chromatogram

| **A.**  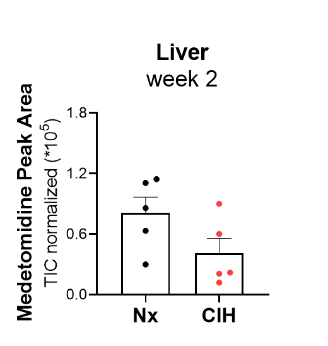 | **B.**  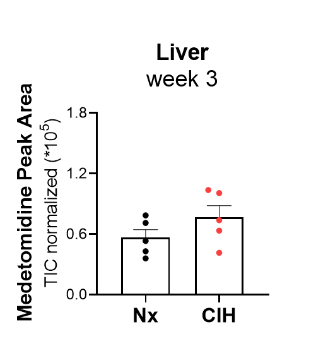 | **C.**  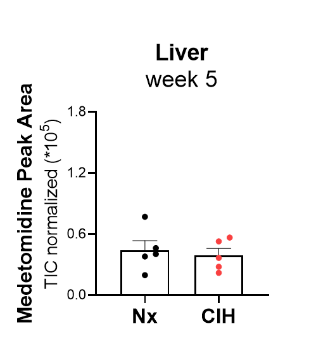 | **D.**  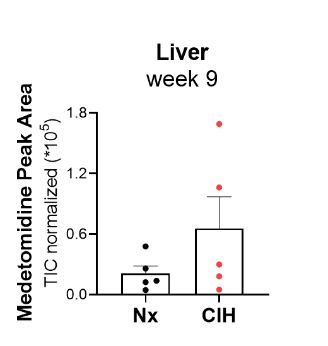 |
| --- | --- | --- | --- |
| **E.**  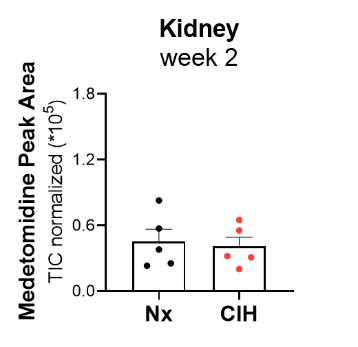 | **F.**  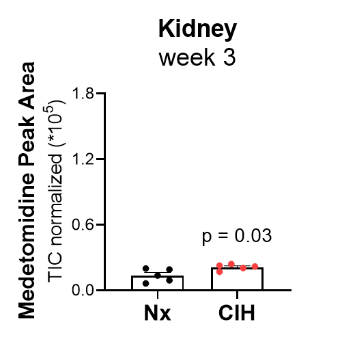 | **G.**  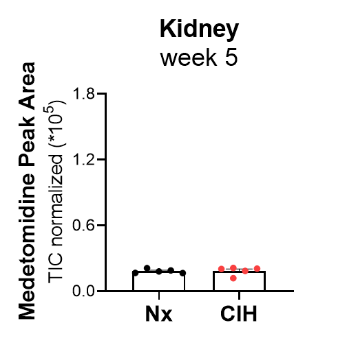 | **H.**  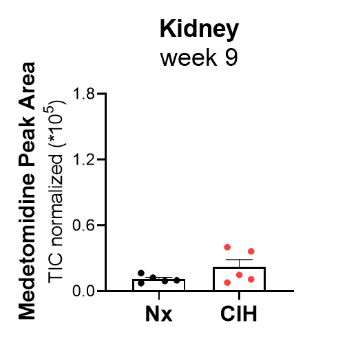 |

**Fig. S4. Medetomidine disposition in liver and kidney tissues**, at **(A)** and **(E)** week 2, **(B)** and **(F)** week 3, **(C)** and **(G)** week 5, and **(D)** and **(H)** week 9 of exposure to chronic intermittent hypoxia (CIH) vs normoxia (Nx) paradigms. The represented *p*-value is derived from unpaired t-test. **AU**, arbitrary units; **TIC**, total ion chromatogram

| **A.**  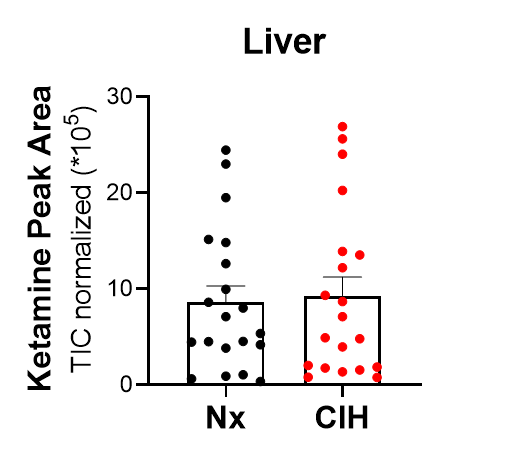 | **B.**  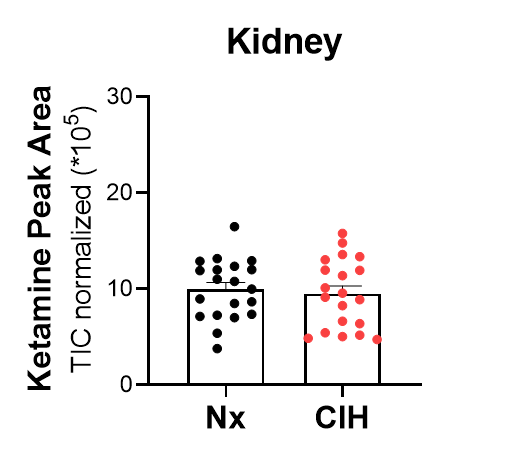 |
| --- | --- |
| **C.**  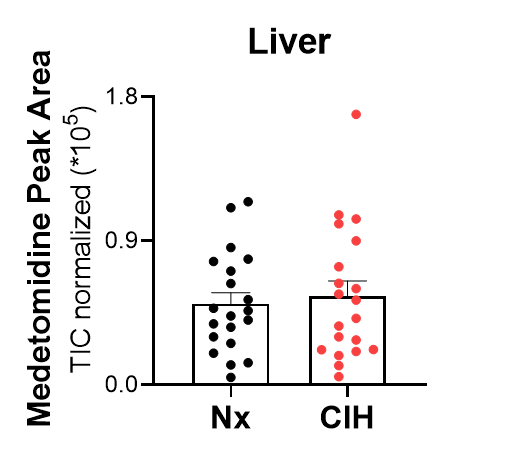 | **D.**  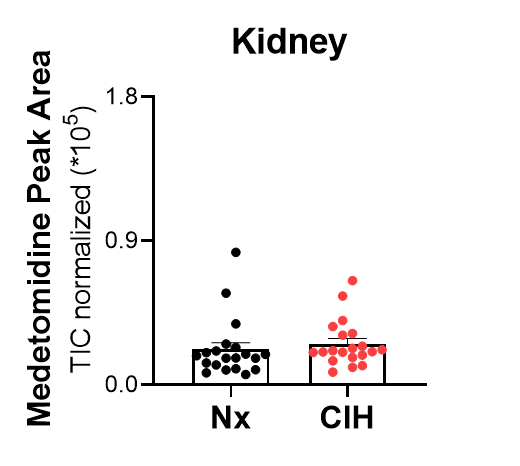 |

**Fig. S5.** **Inter-animal variability in animals exposed to normoxic (Nx) and chronic intermittent hypoxia (CIH) conditions** for 2, 3, 5 and 9 weeks (n=20 per group), for ketamine (A, B) and medetomidine (C and D), in the liver and the kidney


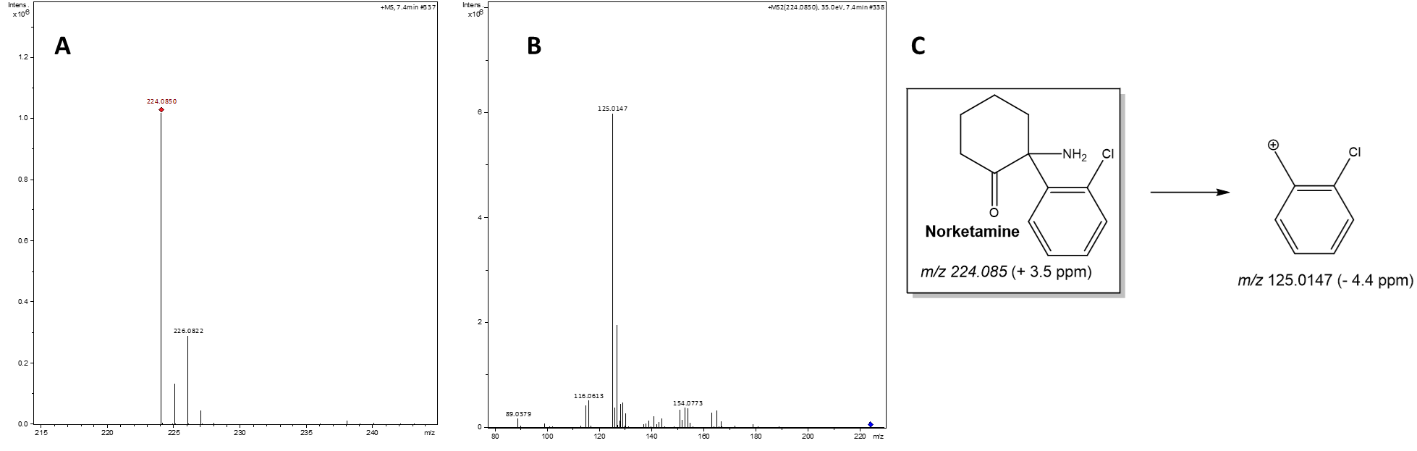
**Fig. S6.** **HRMS-ESI (+) obtained for Norketamine**. **(A)** Full scan mass spectrum; **(B)** tandem mass spectrum and **(C)** potential structures of the main fragment ions


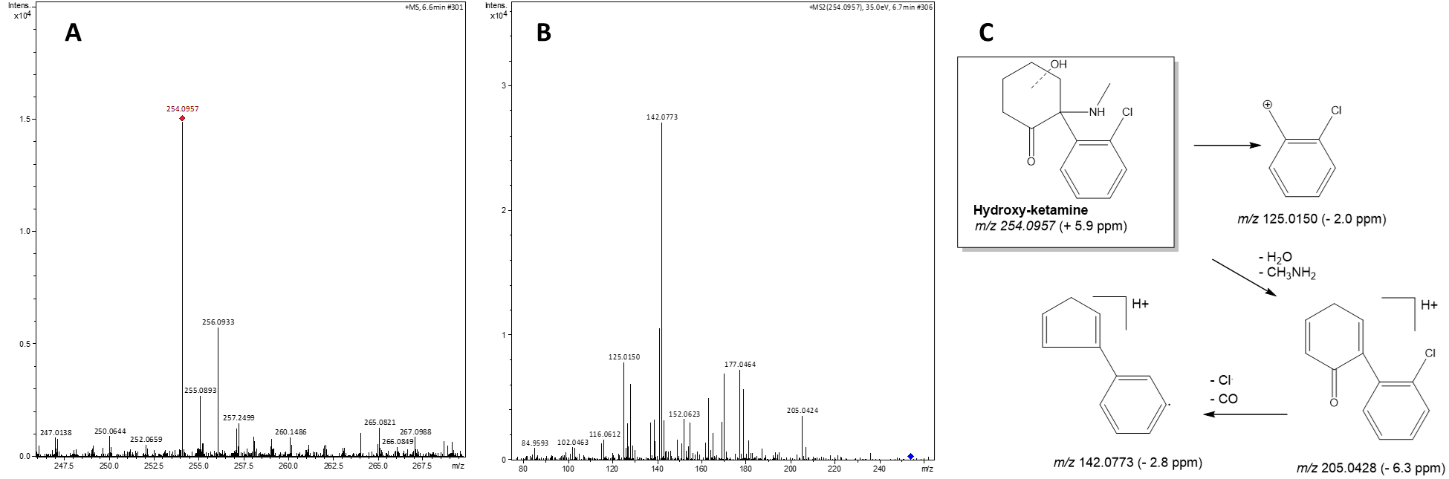
**Fig. S7. HRMS-ESI (+) obtained for Hydroxyketamine.** **(A)** Full scan mass spectrum; **(B)** tandem mass spectrum and **(C)** potential structures of the main fragment ions


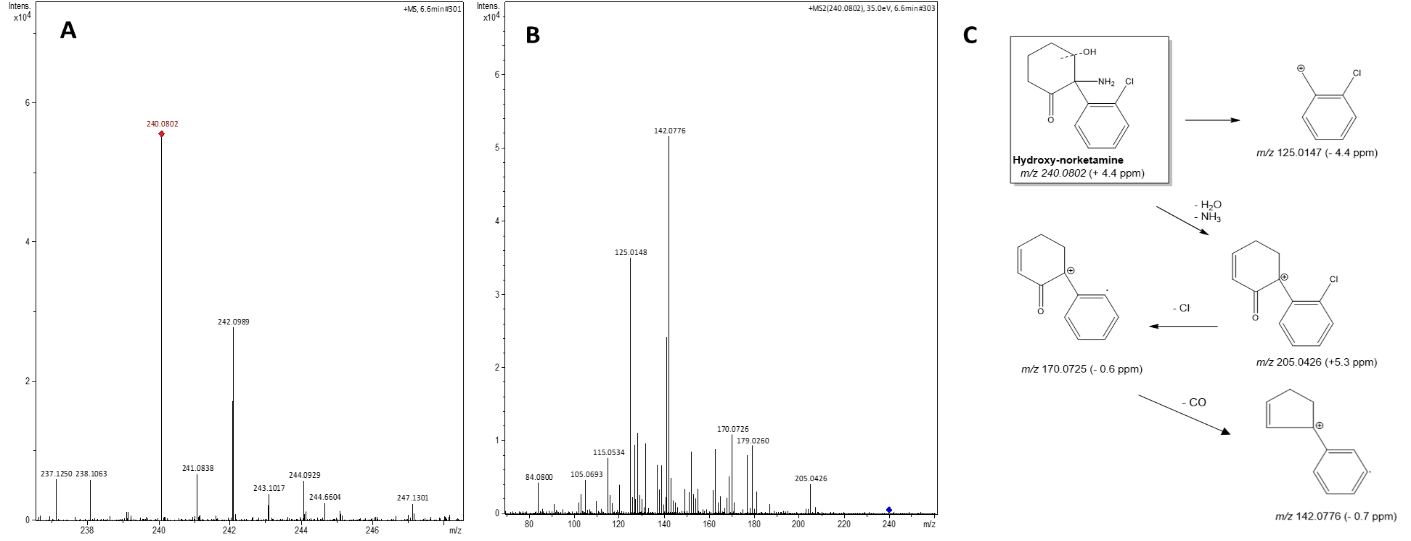
**Fig. S8. HRMS-ESI (+) obtained for Hydroxynorketamine.** **(A)** Full scan mass spectrum; **(B)** tandem mass spectrum and **(C)** potential structures of the main fragment ions


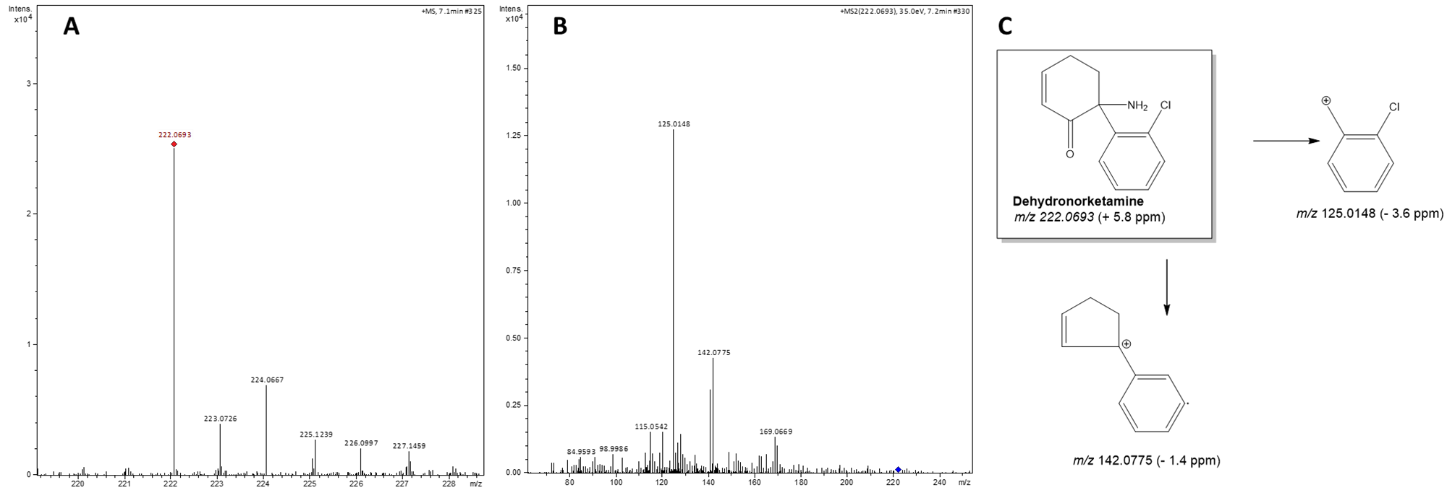
**Fig. S9.** **HRMS-ESI (+) obtained for Dehydronorketamine.** **(A)** Full scan mass spectrum; **(B)** tandem mass spectrum and **(C)** potential structures of the main fragment ions


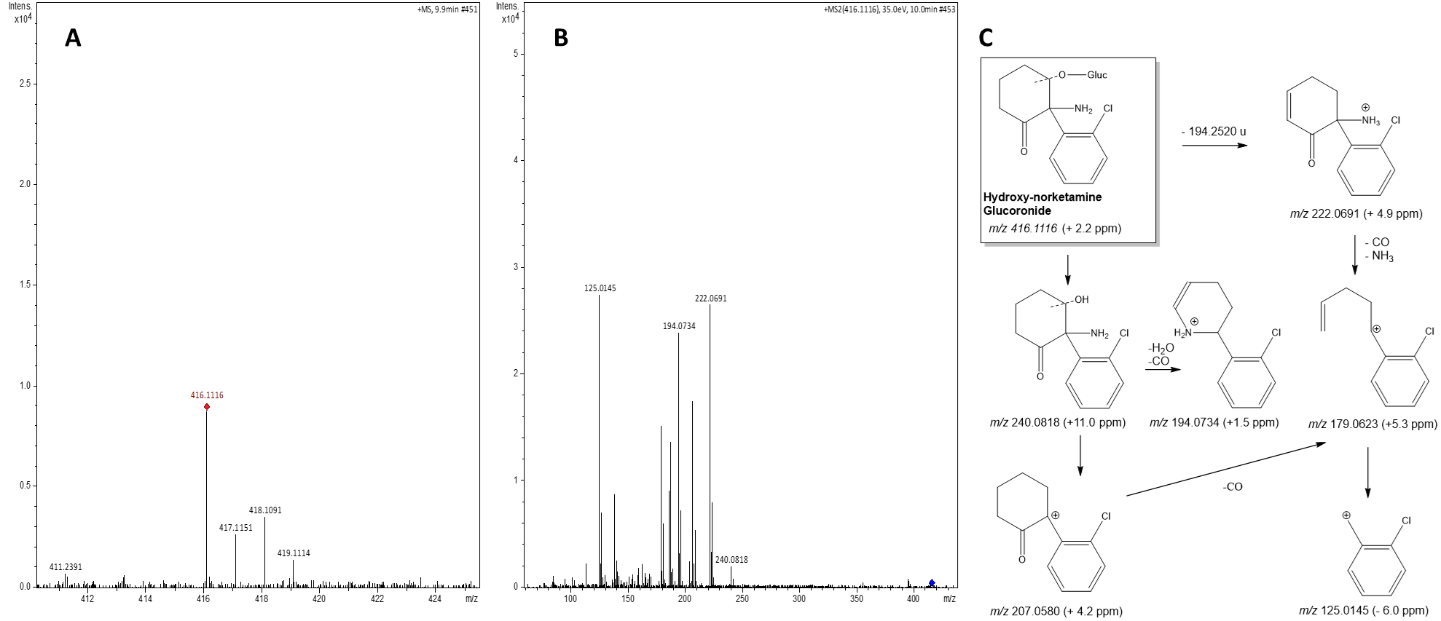
**Fig. S10.** **HRMS-ESI (+) obtained for Hydroxynorketamine glucuronide.** **(A)** Full scan mass spectrum; **(B)** tandem mass spectrum and **(C)** potential structures of the main fragment ions

| A.  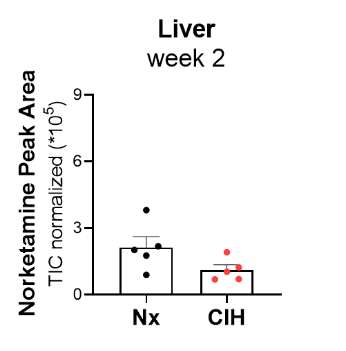 | B.  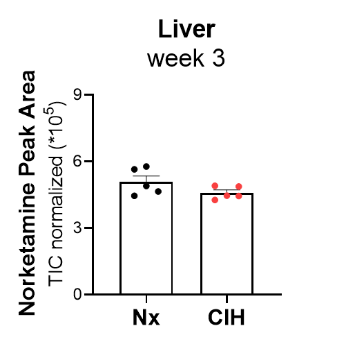 | C.  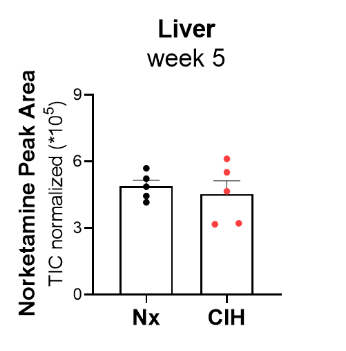 | D.  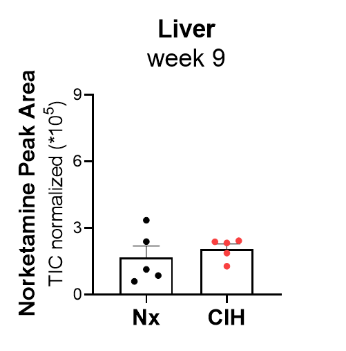 |
| --- | --- | --- | --- |
| E.  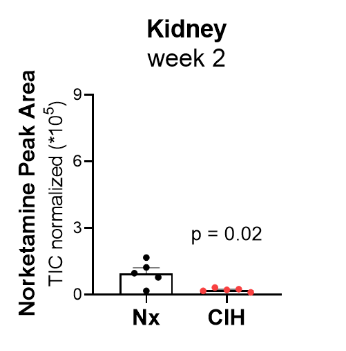 | **F.**  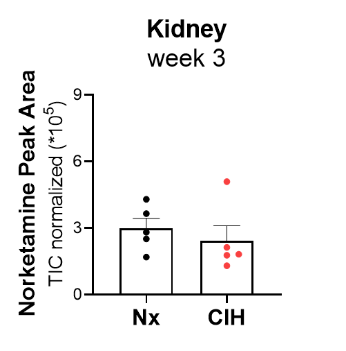 | **G.**  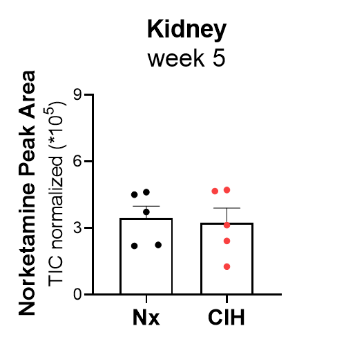 | **H.**  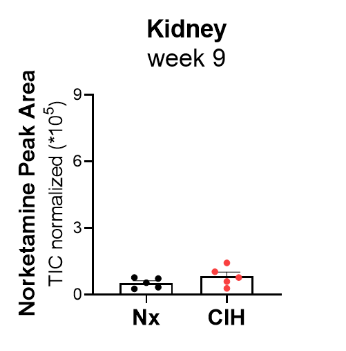 |

**Fig. S11. Norketamine disposition in liver and kidney tissues**, at **(A)** and **(E)** week 2, **(B)** and **(F)** week 3, **(C)** and **(G)** week 5, and **(D)** and **(H)** week 9 of exposure to chronic intermittent hypoxia (CIH) vs normoxia (Nx) paradigms. The represented *p*-value is derived from unpaired t-test. n=5 animals per group. **AU**, arbitrary units; **TIC**, total ion chromatogram

| A.  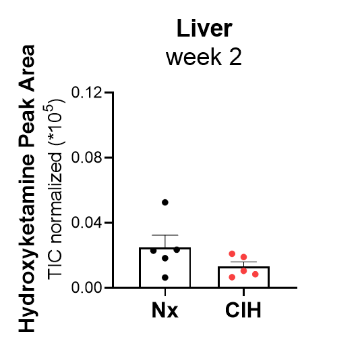 | B.  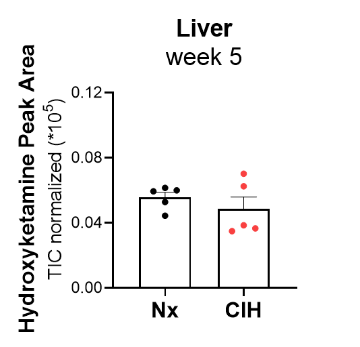 | C.  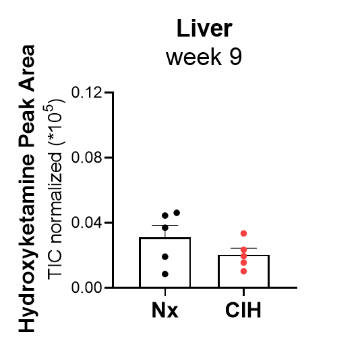 |
| --- | --- | --- |
| D.  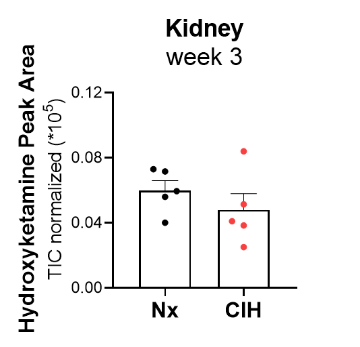 | **E.**  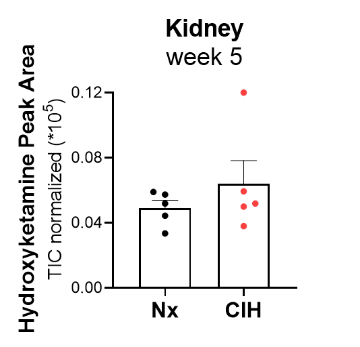 | **F.**  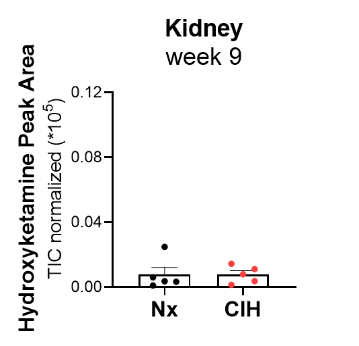 |

**Fig. S12. Hydroxyketamine disposition in liver and kidney tissues,** at **(A)** week 2, **(D)** week 3, **(B)** and **(E)** week 5, and **(C)** and **(F)** week 9 of exposure to chronic intermittent hypoxia (CIH) vs normoxia (Nx) paradigms. The represented *p*-values are derived from unpaired t-tests. n=5 animals per group. **AU**, arbitrary units; **TIC**, total ion chromatogram

| A.  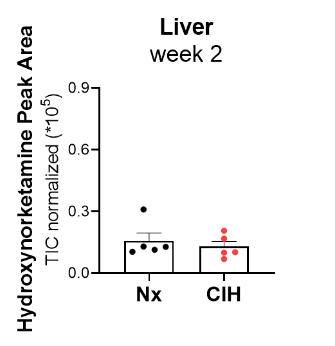 | B.  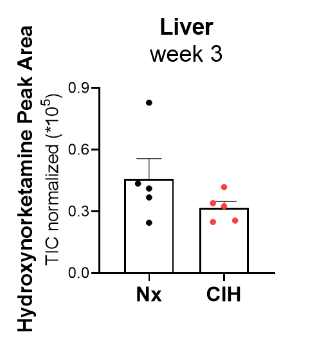 | C.  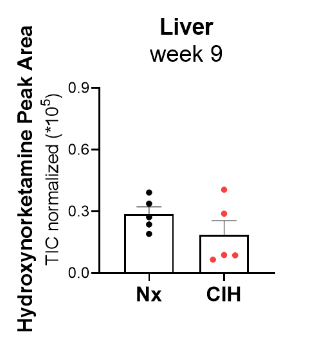 |
| --- | --- | --- |
| D.  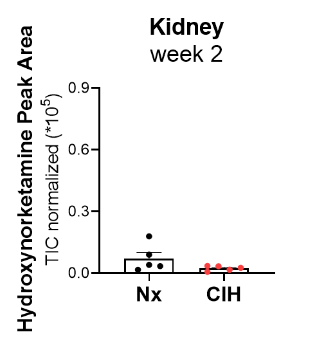 | **E.**  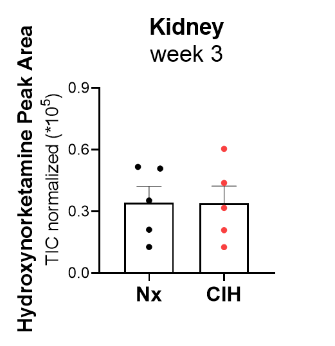 | **F.**  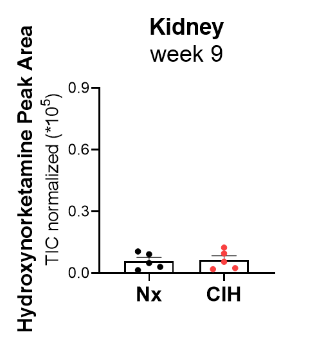 |

**Fig. S13. Hydroxynorketamine disposition in liver and kidney tissues**, at **(A)** and **(D)** week 2, **(B)** and **(E)** week 3, and **(C)** and **(F)** week 9 of exposure to chronic intermittent hypoxia (CIH) vs normoxia (Nx) paradigms. The represented *p*-values are derived from unpaired t-tests. n=5 animals per group. **AU**, arbitrary units; **TIC**, total ion chromatogram

| A.  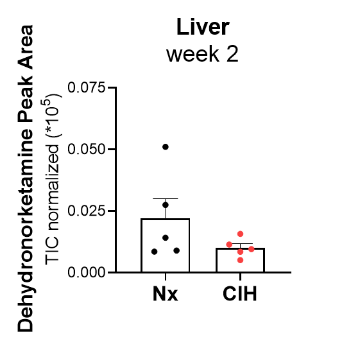 | B.  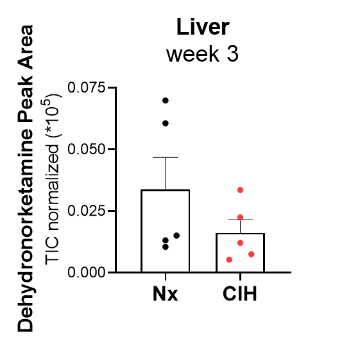 | C.  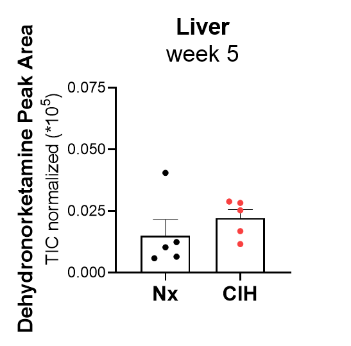 | D.  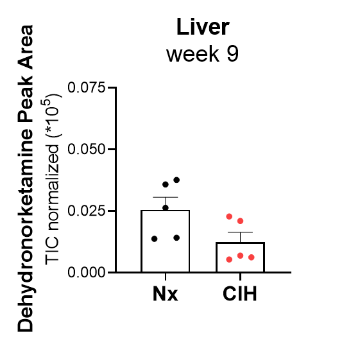 |
| --- | --- | --- | --- |
| E.  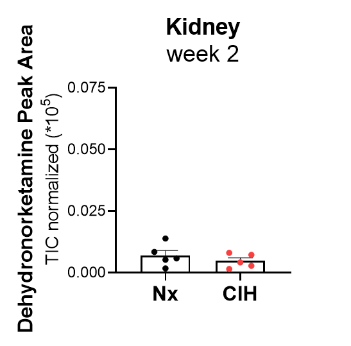 | **F.**  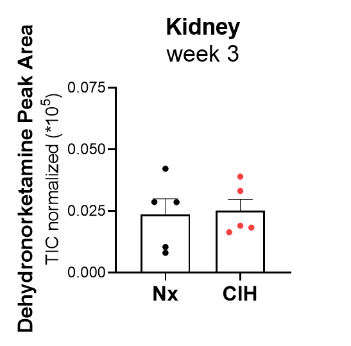 | **G.**  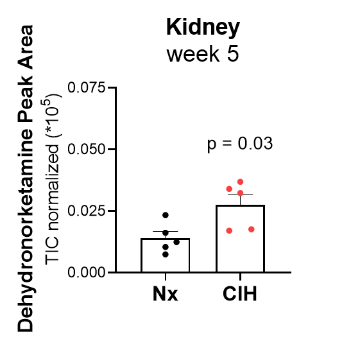 | **H.**  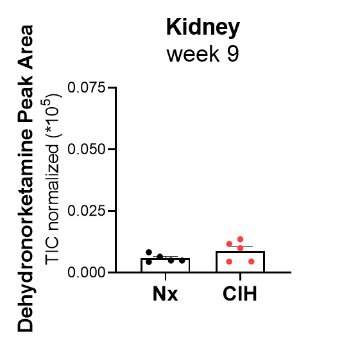 |

**Fig. S14. Dehydronorketamine disposition in liver and kidney tissues**, at **(A)** and **(E)** week 2, **(B)** and **(F)** week 3, **(C)** and **(G)** week 5, and **(D)** and **(H)** week 9 of exposure to chronic intermittent hypoxia (CIH) vs normoxia (Nx) paradigms. The represented *p*-value is derived from unpaired t-test. n=5 animals per group. **AU**, arbitrary units; **TIC**, total ion chromatogram

| A.  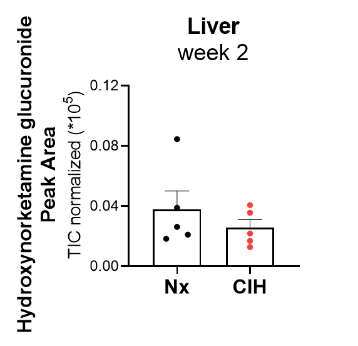 | B.  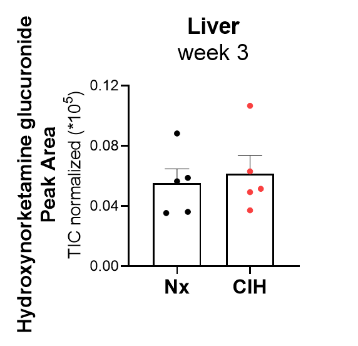 | C.  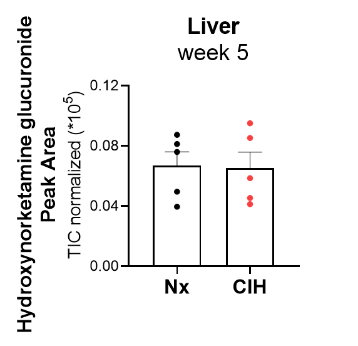 | D.  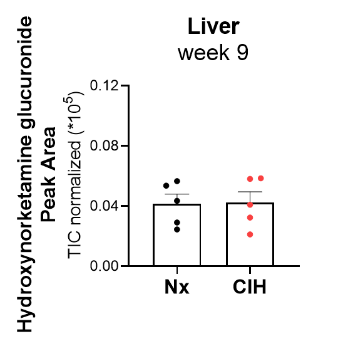 |
| --- | --- | --- | --- |
| E.  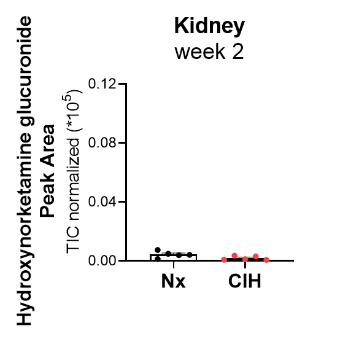 | **F.**  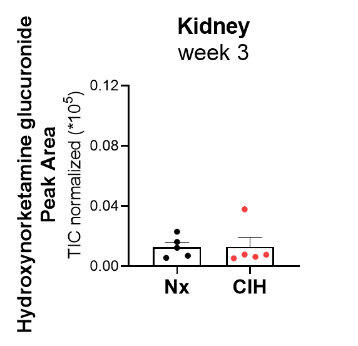 | **G.**  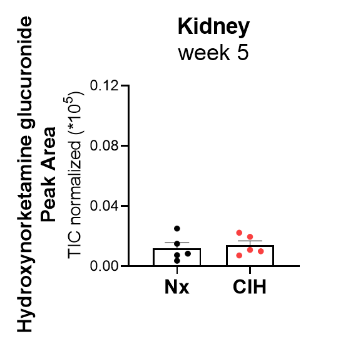 | **H.**  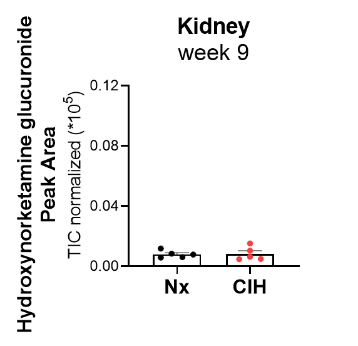 |

**Fig. S15. Hydroxynorketamine glucuronide disposition in liver and kidney tissues**, at **(A)** and **(E)** week 2, **(B)** and **(F)** week 3, **(C)** and **(G)** week 5, and **(D)** and **(H)** week 9 of exposure to chronic intermittent hypoxia (CIH) vs normoxia (Nx) paradigms. n=5 animals per group. **AU**, arbitrary units; **TIC**, total ion chromatogram

| A.  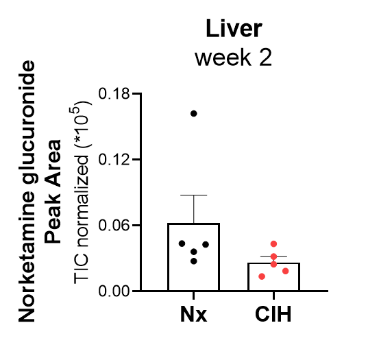 | B.  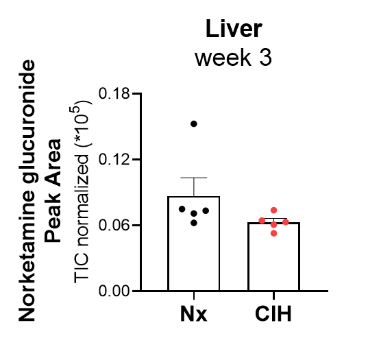 | C.  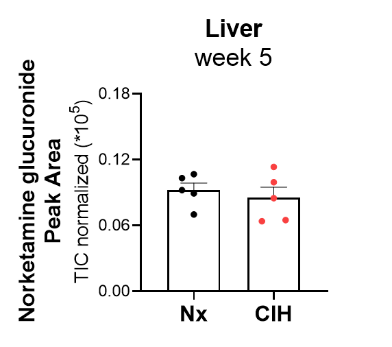 | D.  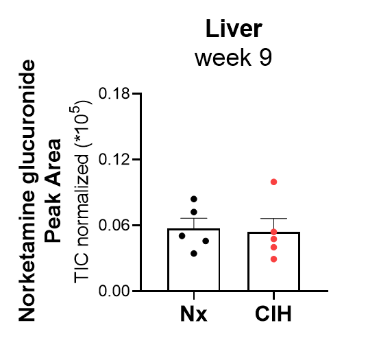 |
| --- | --- | --- | --- |
| E.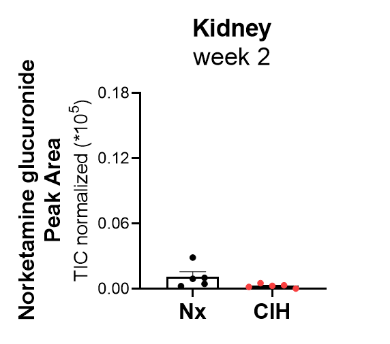 | **F.**  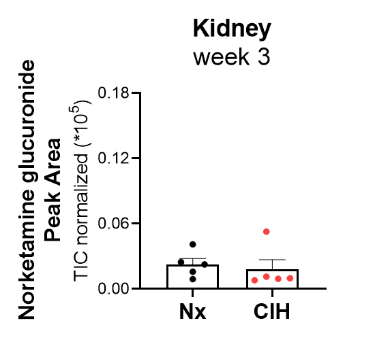 | **G.**  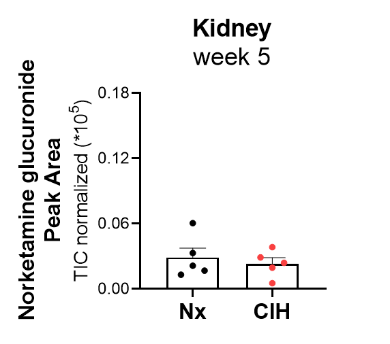 | **H.**  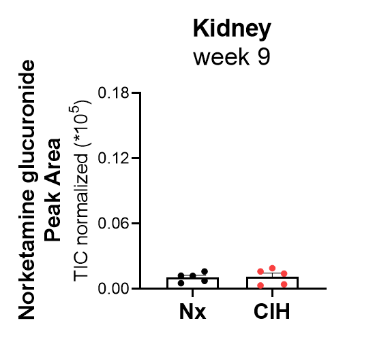 |

**Fig. S16. Norketamine glucuronide disposition in liver and kidney tissues**, at **(A)** and **(E)** week 2, **(B)** and **(F)** week 3, **(C)** and **(G)** week 5, and **(D)** and **(H)** week 9 of exposure to chronic intermittent hypoxia (CIH) vs normoxia (Nx) paradigms. n=5 animals per group. **AU**, arbitrary units; **TIC**, total ion chromatogram

| **A.**  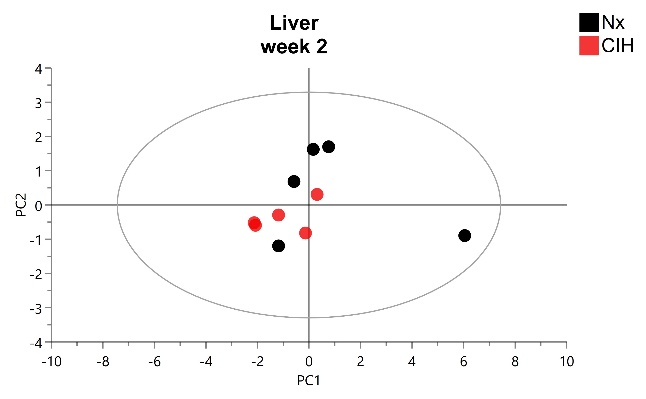 | **B.**  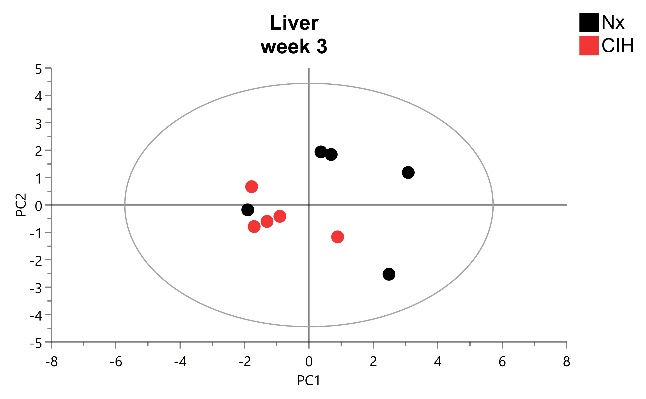 |
| --- | --- |
| **C.**  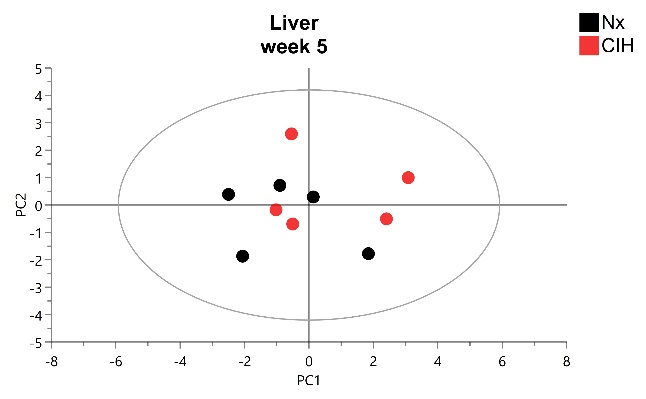 | **D.**  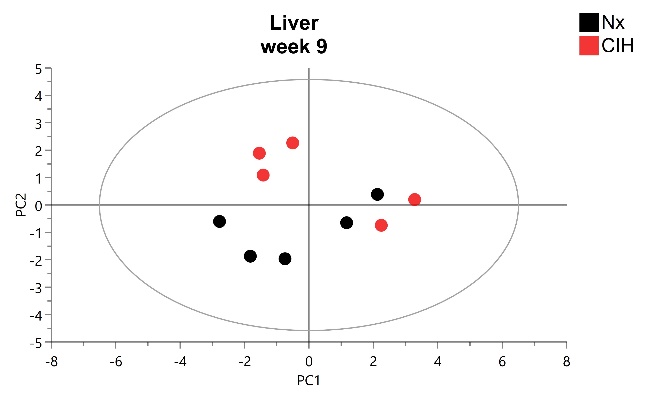 |

**Fig. S17. Profiling of ketamine biotransformation in liver tissues,** at **(A)** week 2, **(B)** week 3 **(C)** week 5, and **(D)** week 9 of exposure to chronic intermittent hypoxia (CIH) in comparison with normoxia (Nx) paradigms. Principal Component Analysis (Score plot); the first two components covered **(A)** 79% and 15%, **(B)** 47% and 28%, **(C)** 50% and 25%, **(D)** 60% and 30% of the variance of the data, respectively. n=5 animals per group. **PC**: Principal Component

| **A.**  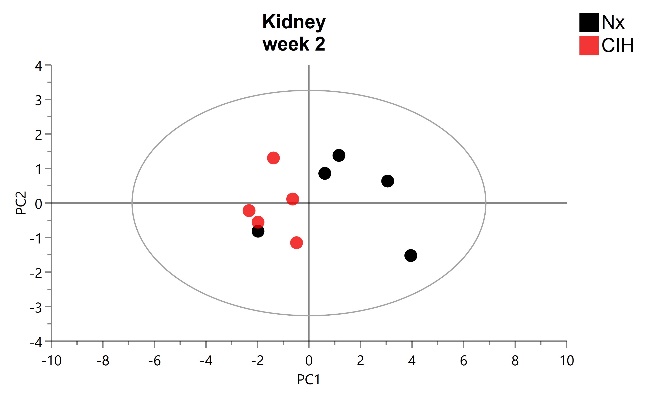 | **B.**  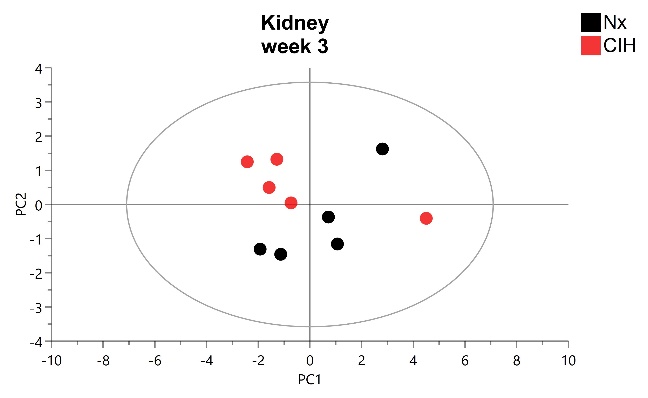 |
| --- | --- |
| **C.**  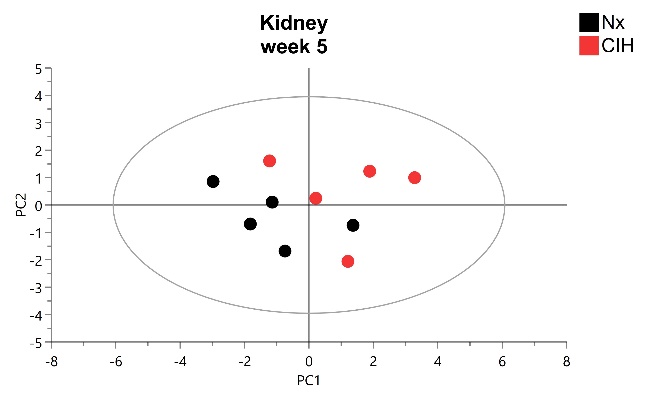 | **D.**  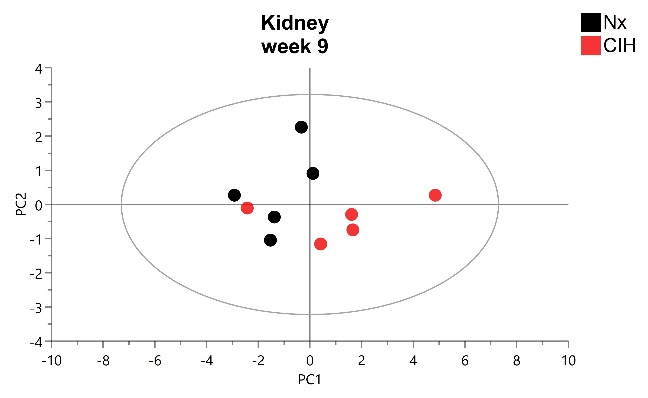 |

**Fig. S18. Profiling of ketamine biotransformation in kidney tissues,** at **(A)** week 2, **(B)** week 3 **(C)** week 5, and **(D)** week 9 of exposure to chronic intermittent hypoxia (CIH) in comparison with normoxia (Nx) paradigms. Principal Component Analysis (Score plot); the first two components covered **(A)** 67% and 15%, **(B)** 72% and 18%, **(C)** 53% and 22%, **(D)** 76% and 15% of the variance of the data, respectively. n=5 animals per group. **PC**: Principal Component

| **A.**  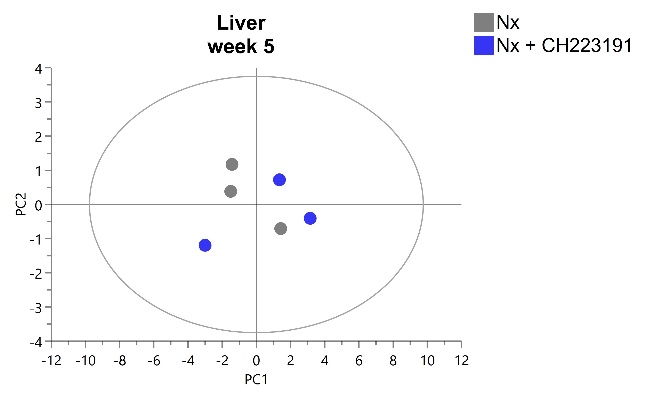 | **B.**  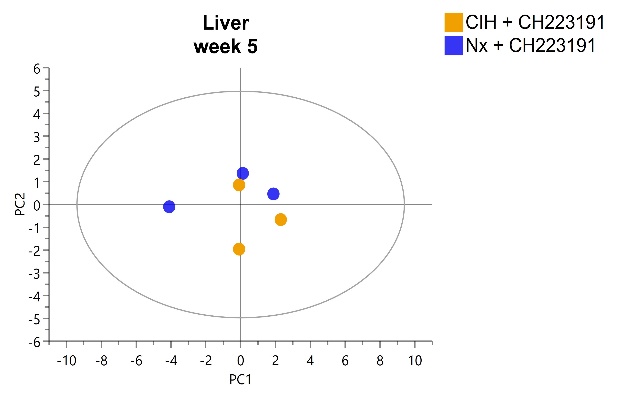 |
| --- | --- |
| **C.**  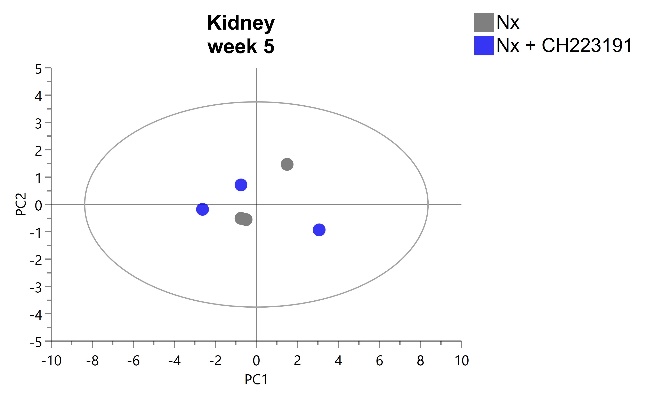 | **D.**  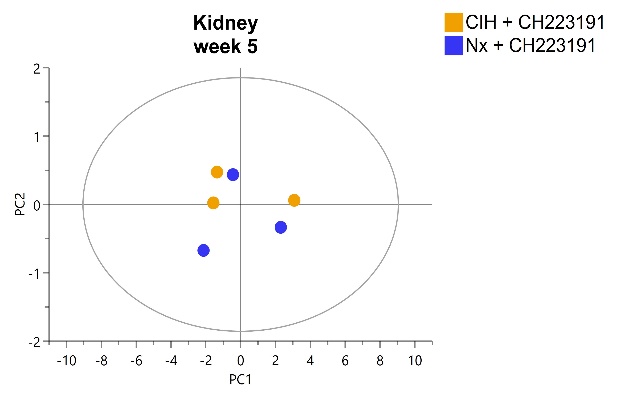 |

**Fig. S19. Profiling of ketamine biotransformation** in **(A, B)** liver and **(C, D)** kidney tissues in animals exposed to normoxia (Nx) conditions or treated with the aryl hydrocarbon receptor (AhR) antagonist CH-223191 under either normoxia (Nx + CH-223191) or chronic intermittent hypoxia (CIH + CH-223191). Principal Component Analysis (Score plot); the first two components covered **(A)** 78% and 12%, **(B)** 73% and 20%, **(C)** 81% and 16%, **(D)** 94% and 4% of the variance of the data, respectively. n=3 animals per group. **PC**: Principal Component

| **A.**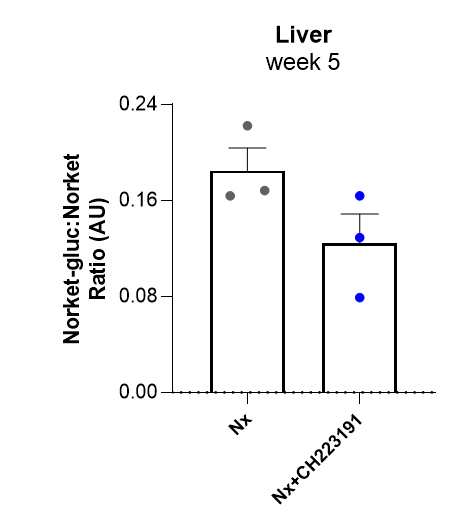 | **B.**  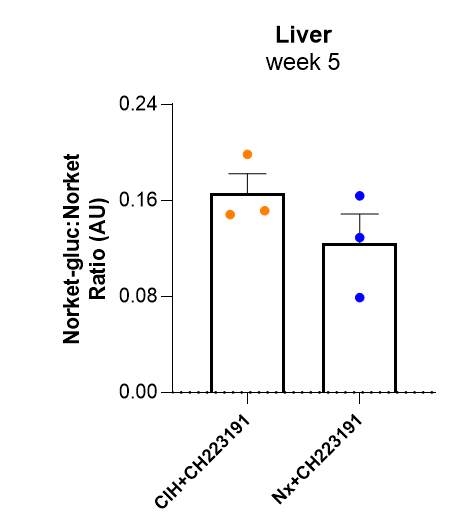 |
| --- | --- |

**Fig. S20. Effect of the AhR antagonist CH-223191 in the liver: metabolite ratios (A)** in Nx and **(B)** in CIH for norketamine (Norket-gluc:Norket). The represented *p*-values are derived from unpaired t-tests. n=3 animals per group. **AU**, arbitrary units
